# Mechanisms to medicines: navigating drug repurposing strategies in Alzheimer’s disease

**DOI:** 10.3389/fnagi.2025.1676065

**Published:** 2026-01-20

**Authors:** Sara Akhtar Khan, Khushi Raza, Prachi Tiwari, Mohamed El-Tanani, Syed Arman Rabbani, Suhel Parvez

**Affiliations:** 1 Department of Toxicology, School of Chemical and Life Sciences, Jamia Hamdard, New Delhi, India; 2 Department of Physiotherapy, School of Nursing Sciences and Allied Health, Jamia Hamdard, New Delhi, India; 3 RAK College of Pharmacy, RAK Medical and Health Sciences University, Ras Al-Khaimah, United Arab Emirates; 4 Department of Clinical Pharmacy and Pharmacology, RAK College of Pharmacy, RAK Medical and Health Sciences University, Ras Al-Khaimah, United Arab Emirates

**Keywords:** Alzheimer’s disease, pathophysiology, drug repurposing, aging, network biology, therapeutic strategies

## Abstract

Alzheimer’s disease (AD) represents a continuously advancing neurodegenerative condition distinguished by the unremitting deterioration of cognitive abilities and memory impairment, which significantly hampers daily functioning of life. In the absence of disease modifying treatments, it continues to pose a significant global challenge. Though symptomatic treatment exists, the inherent complexity involved with AD pathogenesis related to Aβ plaques, neurofibrillary tangles, neuroinflammation, oxidative stress, etc. poses a tremendous challenge to developing drugs. With the incidence of AD increasing yearly globally, research into already existing pharmacological agents has the potential to uncover a brighter future for breakthroughs in treatment strategy. A primary strategy to accelerate the development of AD therapies is drug repurposing: determining a new use for an existing known medication. Following innovative approaches like high-throughput screening, AI-based techniques, a number of classes of drugs originally designed for other diseases are now being tested to modulate the complex pathology mechanisms in AD. This review focuses on the therapeutic promise of drug repurposing as adjunctive to the much-needed renaissance in AD therapies. The review continues to focus on some promising repurposed drug candidates, methodologies applied, and the evaluation of the present status of drugs in the clinic. Apart from the information regarding mechanisms involved in AD, this review also complements case studies, challenges, and limitations along with the various drug repurposing strategies for AD. By understanding and harnessing the potential of existing pharmacological agents, we can expand therapeutic options and improve patient outcomes.

## 1 Introduction

**FIGURE 1:**
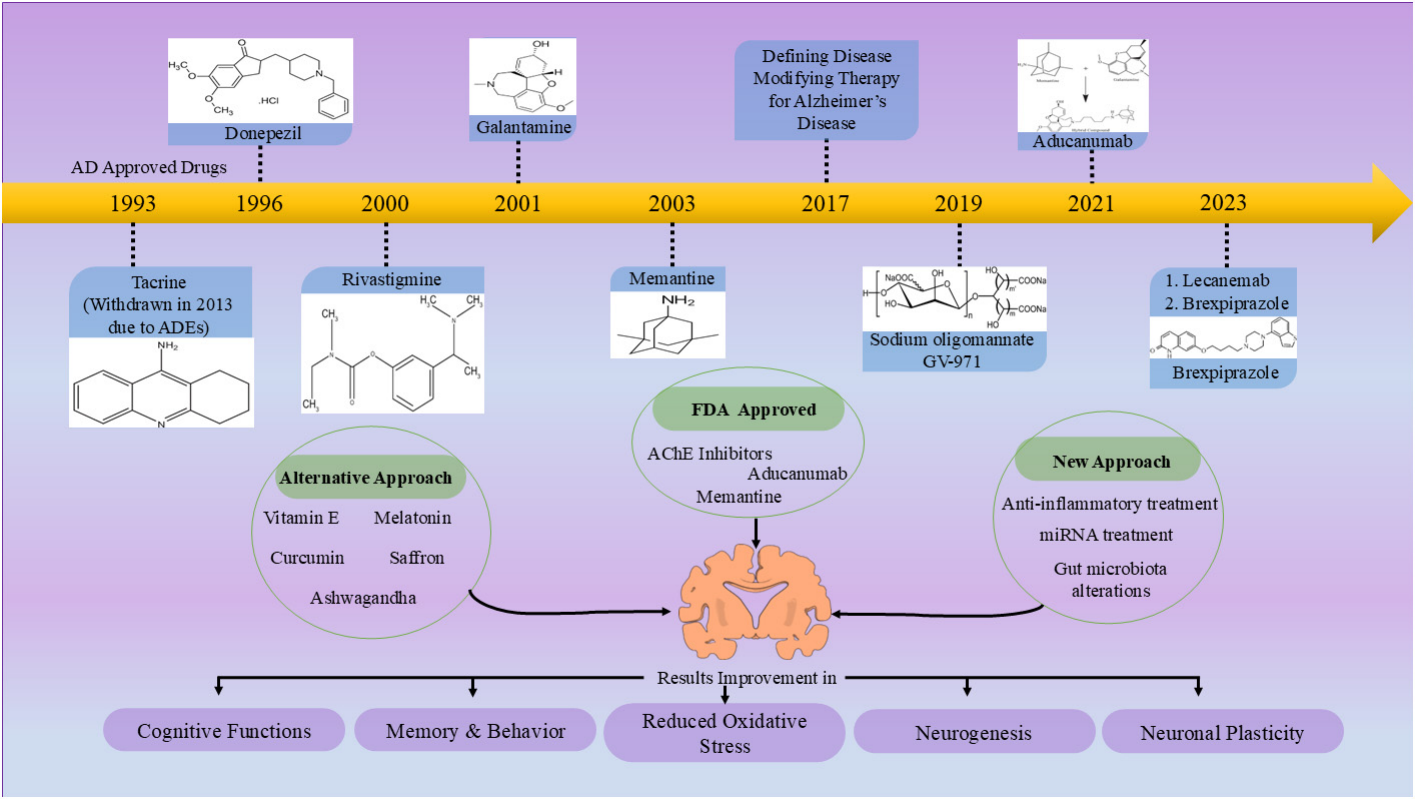
Chronological order of approved drugs and various approaches to treat Alzheimer’s disease (AD). The diagram depicts the time-evolving approval of symptomatic drugs like cholinesterase inhibitors and NMDA receptor antagonists and emphasizes recent advances in disease-modifying drugs like monoclonal antibodies against Aβ for the treatment of AD. Apart from these established treatments, the figure reflects on other potential options such as vitamin E, melatonin, ashwagandha, saffron, and curcumin and newer approaches such as anti-inflammatory treatment, miRNA-based treatment, and gut microbiota modulation. All of them have proven beneficial in terms of improvements in cognitive functions, memory, behavior, neurogenesis, neuronal plasticity, and reduction in oxidative stress. This overview summarizes the multiple therapeutic agents being assessed for their value for AD, from conventional medicinal agents to novel and complementary drugs, all aimed at ameliorating the multifactorial pathologies of this disease toward better outcomes for patients.

Alzheimer’s disease (AD) is a neurodegenerative disorder characterized by gradual memory loss and cognitive decline. It is the most common cause of dementia, affecting millions globally (Budson and Solomon, 2011). The clinical diagnosis of AD is the result of the culmination of a decade-long process of a disease that is silent, with degradation of brain structure and function taking place 20–30 years before clinical manifestation appear (Aisen et al., 2017). At present, there is no definitive cure for the condition; however, several therapies can assist in managing the symptoms. U.S. Food and Drug Administration (FDA) has approved Seven drugs, including two amyloid beta- directed monoclonal antibodies named lecanemab and aducanumab, a glutamate regulator or more specifically NMDA antagonist, memantine, three cholinesterase inhibitors named as donepezil, rivastigmine, and galantamine and a combination of a cholinesterase inhibitor and glutamate regulator called donepezil and memantine, to mitigate the progression of AD (Cummings et al., 2019; Grabowska et al., 2023; Pan et al., 2024; Figure 1). The development of novel therapeutics for AD has not advanced much. Also, the development of new AD drugs has encountered numerous challenges like high costs and lengthy timelines, along with elevated failure rates in preclinical and clinical trials. Developing new treatments for AD has become extremely difficult due to the intricate and not well understood mechanisms of the disease, as well as its varied clinical manifestations and associated comorbid conditions (Cummings et al., 2014; Yiannopoulou et al., 2019). In light of these challenges, researchers have started to explore alternative and new strategies to identify potential therapies for AD (Figure 1). Drug repurposing seeks to discover existing medications that can be redirected for the treatment of AD. Over the last decade, many drugs have been suggested for repurposing in the context of AD, employing a variety of approaches. This strategy offers distinct advantages, including minimized development cost, shorter timelines and already established safety profiles (Pushpakom et al., 2019). In AD, where *de novo* drug discovery has faced exceptionally high failure rates, drug repurposing provides a realistic alternative by leveraging compounds already known to target mechanism relevant to AD, for example, neuroinflammation, mitochondrial dysfunction and metabolic dysregulation (Ballard et al., 2020; Grabowska et al., 2023). Moreover, advances in computational biology and AI-based prediction tools now facilitate more systematic identification of repurposing compounds, thereby reducing reliance on serendipity (Xu Y. et al., 2021; Yan et al., 2024). This review aims to explore the impact of drug repurposing, the current landscape of repurposed drugs and various innovative approaches via which we can target the various frontiers in the treatment of AD. Nevertheless, we have also discussed inherent limitations, including translational challenges, diseases heterogeneity and the need of rigorous clinical validation.

## 2 Alzheimer’s disease

Alzheimer’s disease is a complicated multifactorial neurodegenerative condition that is the major cause of senile dementia, behavior alterations which significantly impairs day-to-day functioning (Firmani et al., 2024; Tenchov et al., 2024; Muraleva et al., 2025). Usually, AD initiates gradually and progresses over time, eventually resulting in significant impairments in memory, reasoning, judgment, and language abilities (DeTure and Dickson, 2019; Budson and Solomon, 2022). It markedly decreases life expectancy and is ultimately a lethal type of dementia (Alzheimer’s and Dementia, 2023). AD is a growing global health concern that affects around 50 million people globally. It is responsible for 60–70% of instances, most of which involve people over 65 (Alzheimer’s and Dementia, 2023). As life expectancy increases, the number of individuals affected by AD is projected to grow (World Health Organization [WHO], 2023; National Institute on Aging, 2024). Consequently, it is anticipated that by 2050 AD may affect approximately 152 million people worldwide, presenting a rapidly escalating issue concerning costs, mortality rates and overall burden (Cummings et al., 2019). Regardless of years of experimental advancements, the management of AD continues to be inadequate, highlighting the need for a more precise understanding of potential therapeutic targets and signaling pathways (Pan et al., 2024).

AD is distinguished by two major pathological features, (a) the accumulation of senile plagues made up of fibrils from the amyloid beta or Aβ peptide (Pichet Binette et al., 2024) and (b) the presence of intracellular neurofibrillary tangles (NFTs), which arise from the hyperphosphorylation of twisted filaments of the tau protein (Shefa et al., 2019; Kumar and Bansal, 2022). The formation of Aβ plaques starts when β-secretase and γ-secretase abnormally cleave the APP, resulting in the extracellular buildup of Aβ, particularly the Aβ_42_ form (Pichet Binette et al., 2024). As AD worsens, memory loss gets more severe and linguistic problems become more pronounced. People start having more difficulties in decision- making and problem-solving activities, exhibiting poor judgment in everyday situations. Agitation, aggression, wandering or social disengagement are the examples of behavioral signs that could worsen. Also, physical symptoms like difficulty walking, swallowing, and performing self-care tasks become difficult too. AD patients become more reliant on others for everyday tasks as the condition progresses (Alzheimer’s and Dementia, 2023). In more advanced stages, individuals might no longer be able to recognize family members, communicate or even manage their movements. They often require continuous assistance in nursing homes or at home with the help of caregivers. Malnutrition, infections and falls are among the physical issues that worsen and further deteriorate health (Alzheimer’s and Dementia, 2023). The most significant risk factor for AD is advanced age, with the majority of cases appearing in those over 65. Additional risk factors include genetic predisposition, family history and lifestyle elements like cardiovascular health and educational background (Murphy and Levine, 2010).

The diagnosis of AD involves recognizing its characteristics pathological features and is generally based on a blend of medical history, cognitive evaluations, neurological examinations and excluding other potential causes of the symptoms (Arvanitakis et al., 2019; Deture and Dickson, 2019; Epelbaum and Cacciamani, 2023). Although there is currently no cure for AD, various medications and non-pharmacological approaches can assist in slowing the progression and managing symptoms, thereby enhancing the patients’ quality of life. Services that provide support such as counseling, support groups and respite care, are essential for coping with the effects of AD on daily routines. Caregivers experience a considerable burden, leading to emotional, physical and financial strain. The economic ramifications of AD are significant, encompassing direct medical expenses, indirect costs as well as lost productivity (Ameri et al., 2024).

## 3 Mechanisms involved in AD

**FIGURE 2:**
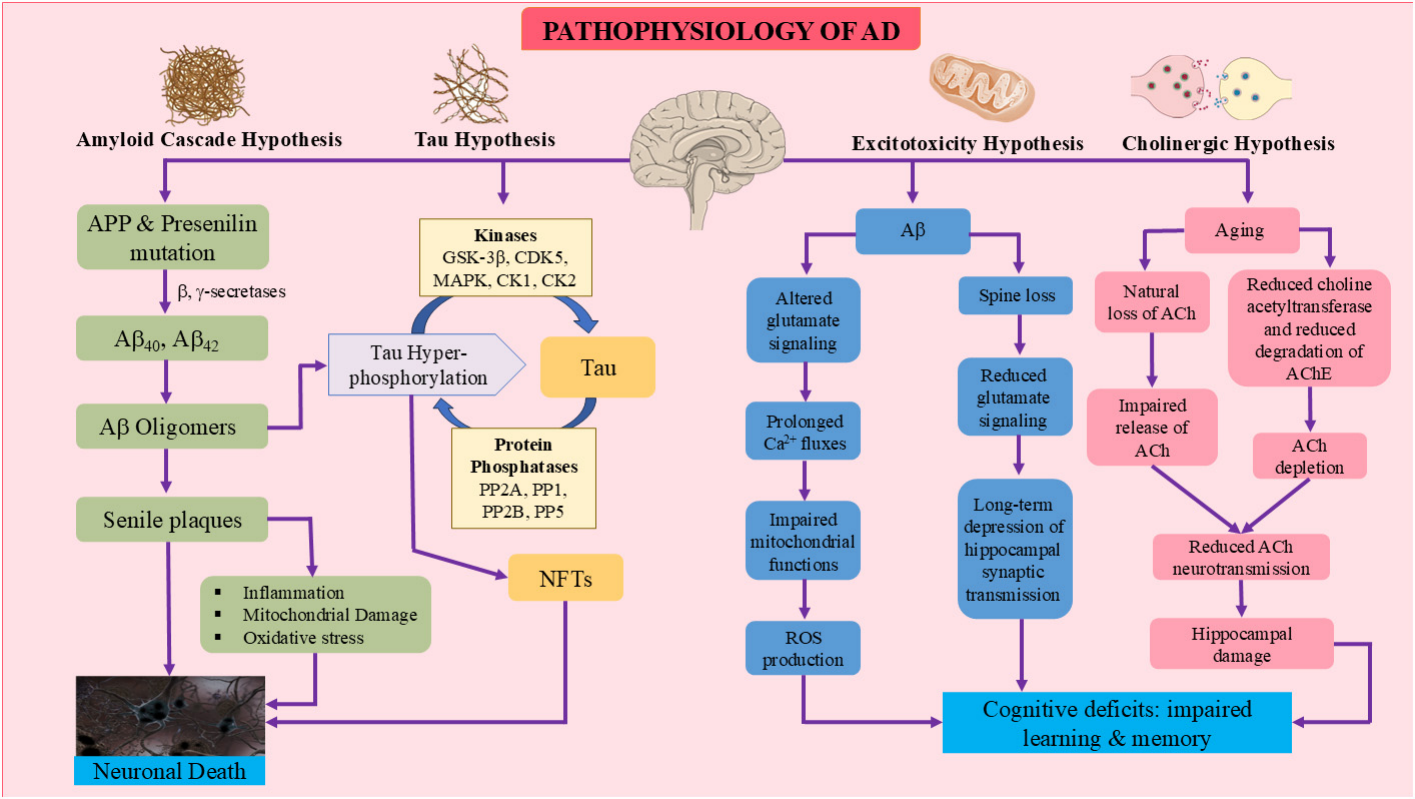
Pathophysiological mechanisms in Alzheimer’s disease (AD). The diagrammatic flowchart shows the amyloid cascade, tau pathology, excitotoxicity, and cholinergic dysfunction, demonstrating how these interrelated pathways lead to AD progression. In the amyloid cascade hypothesis, APP and presenilin mutations cause elevated β- and γ-secretase activity, leading to the generation of Aβ_40_ and Aβ_42_. Aβ aggregation leads to the formation of soluble oligomers, which then aggregate to form senile plaques. Aβ oligomers cause tau hyperphosphorylation via kinases; phosphatases reverse this process. Hyperphosphorylated tau gives rise to paired helical filaments, which aggregate and become neurofibrillary tangles (NFTs). Senile plaques lead to mitochondrial impairment, oxidative stress, and inflammatory reactions, leading to neuronal death. The excitotoxicity process is due to disrupted calcium homeostasis and glutamate transmission, while the cholinergic hypothesis, which are largely due to aging, encompass decreased acetylcholine levels, resulting in hippocampal injury, generation of reactive oxygen species (ROS), synaptic dysfunction, loss of memory, and overall cognitive impairment.

The mechanisms involved in AD pathophysiology are explained in the subsections. Moreover, the interrelated pathways that link amyloid accumulation, tau hyperphosphorylation, excitotoxicity and cholinergic dysfunction to neuronal death are illustrated in Figure 2. These mechanisms demonstrate how Aβ oligomers trigger tau phosphorylation via kinases including GSK-3β and CDK5, eventually leading to neurofibrillary tangle formation and cognitive decline (De Strooper et al., 2010; O’Brien and Wong, 2011; Hampel et al., 2021; Pichet Binette et al., 2024).

### 3.1 Amyloid hypothesis

The amyloid hypothesis, also known as APP hypothesis, proposes that abnormal APP processing plays an important role in the pathogenesis of AD (Figure 2). According to this hypothesis, neurodegeneration is primarily caused by the production and deposition of amyloid β plaques in the brain (O’Brien and Wong, 2011). Transmembrane protein APP is abundantly expressed in the brain and is mostly processed by the non-amyloidogenic and amyloidogenic pathways in normal circumstances (Hampel et al., 2021). As mentioned earlier, the plaques are produced by the enzymatic cleavage of APP, with α- secretase producing a C-terminal fragments and a soluble APPα. Because this process stops Aβ peptides from forming, it is thought to be neuroprotective. On the other hand, BACE1 also known as β- secretase and γ- secretase sequentially cleave APP in amyloidogenic pathway to produce Aβ peptides, specifically Aβ_40_ and Aβ_42_ (De Strooper et al., 2010). The enhanced ability to aggregate and increased neurotoxicity of Aβ_42_ make it of particular interest. These peptides have the ability to assemble into oligomers, protofibrils, and amyloid fibrils, which ultimately cause extracellular amyloid plaques to develop (Song et al., 2022). Aggregated Aβ species build up and are thought to set off a series of pathogenic events that include neuroinflammation, oxidative stress, synaptic dysfunction, and disturbance of calcium homeostasis. These mechanisms conjointly lead to neuronal damage and cell death, the latter being expressed as cognitive impairment and memory loss: the major features of AD (Azargoonjahromi, 2024; Figure 2).

Recent studies have demonstrated that massive Aβ accumulation significantly impairs neuronal integrity through impairment of synaptic function as well as through disruption of the neuronal signaling pathways. The interference of Aβ peptides with synaptic functionality leads to deficits in learning and memory. Moreover, Aβ causes neuronal cell death due to its disruption of calcium homeostasis (Hardy and Higgins, 1992; Koudinov and Berezov, 2004; Grienberger et al., 2012; Sadigh-Eteghad et al., 2014; Zhang Y. et al., 2023). Genetic studies revealed that the familial variants of AD (FAD) are caused by point mutations in APP, PSEN1, PSEN2 or ApoE4 genes can lead to the increased aggregation of Aβ_1–42_ (Lanoiselée et al., 2017; Arber et al., 2021). In addition, quantitative changes in neuropeptide expression, for example, a decrease in corticotropin releasing hormone (CRH), somatostatin and neuropeptide Y, an increase in angiotensin II, etc., also affect the process of forming amyloid plaques (Futch et al., 2017; Chen et al., 2019; Gouveia et al., 2022; Williams et al., 2023). Early onset of AD is caused by these mutations, which also change Aβ_42_ to Aβ_40_ and increase Aβ production particularly Aβ_42_ (Zhang et al., 2018). The mutations enhance the aggregation and generation of Aβ by disrupting the balance between the non-amyloidogenic and amyloidogenic processing of APP (Kim and Hecht, 2008). This genetic evidence forms the basis for Aβ role in AD pathogenesis, verifying amyloid hypothesis. While making tremendous contributions to progress toward understanding AD and providing a direction for most drug development, the importance of appreciation that AD also is a complex disorder should also be noted. Degeneration and death of neurons are also promoted through NFTs (Cras et al., 1995). Activation of glial cells as a response to Aβ aggregation perpetuates neuronal injury through neuroinflammation (Al-Ghraiybah et al., 2022). Still, the amyloid hypothesis is somehow controversial, since therapies targeting the formation of amyloid plaques did not result in recurrent reversal of cognitive decline (Morris et al., 2014). Current research involves therapeutic targets beyond amyloid, tau proteins, inflammation, and oxidative stress. The amyloid hypothesis informs molecular mechanisms by which AD develops through aberrant APP processing and resultant neurotoxic Aβ peptides.

### 3.2 Tau hypothesis

Tau hypothesis, which is well-established in the field of AD research, claims that abnormal protein aggregation, and hyperphosphorylation of tau microtubule-associated proteins, is the primary cause for development of the disease (Figure 2). Tau proteins are neural proteins associated with microtubules that contain a microtubule binding domain essential for preserving the integrity of the cytoskeleton (Wolfe, 2012; Arnsten et al., 2021). It is necessary to preserve microtubule stability, which is necessary for neurons’ structural integrity and transport processes. Tau, under normal physiological conditions, will preferentially bind with microtubules, which promotes their assembly and stability. Under AD conditions, it is subjected to abnormal phosphorylation modifications in which the highest event noted is hyperphosphorylation that leads to tau detaching from the microtubules. The process causes a kind of intracellular disturbance of flow and striking disruption of networks of microtubules (Medeiros et al., 2010; Barbier et al., 2019). The dysregulation of several kinases and phosphatases leads to the hyperphosphorylation of tau (Basheer et al., 2023). Due to its decreased affinity for microtubules, hyperphosphorylated tau causes microtubule disintegration and impairs intracellular transport. Moreover, hyperphosphorylated tau is more likely to form NFTs and paired helical filaments (PHFs) by self-assembly (Mietelska-Porowska et al., 2014; Alavi Naini and Soussi-Yanicostas, 2015). NFTs cause the brain’s memory and cognitive centers to deteriorate as they proliferate throughout the body (Nelson et al., 2009).

Another study suggests that the binding of tau with microtubules is influenced by the phosphorylation of serine/threonine residues by kinases such as FYN kinase, GSK3β, CDK5 (Chakraborty et al., 2023). CDK5 may play a role in the development of NFTs. Aβ triggers calpain activation, disrupts p35, and, due to an overload of calcium in the cytosol, p35 is cleaved into p25. This leads to the overactivation of CDK5 and results in the hyperphosphorylation of tau, reducing its affinity for microtubules and causing the formation of NFTs, which accumulate in the cytosol and impact normal cellular activities such as synaptic transmission, axonal transport, and signal transduction (Pao and Tsai, 2021).

Novel research reports that tau pathology may spread in the brain just like in prion diseases (Mudher et al., 2017). Localized neurons can uptake pathological forms of tau aggregate formed by the insulted neurons, thus leading to proper tau protein misfolding and aggregation in the recipient cells (Wolfe, 2012). The tau pathology’s prion-like proliferation promotes the NFTs’ dispersion throughout the many regions of the brain in AD. This mode of dissemination emphasizes the progressive nature of tau pathology and its contribution to the extensive neurodegeneration observed in AD (Zhang H. et al., 2021). Tau hypothesis is of enormous importance in the study of AD; it supports the amyloid hypothesis, because aggravation of Aβ toxicity by tau pathology starts a vicious cycle of neurodegeneration and cognitive impairment. Tau-Aβ interaction points to a very complex, multifaceted nature of AD; here, several degenerative processes converge to drive forward the growth of the disease. The tau theory also gets further evidence from genetic studies. Tauopathies are a family of neurodegenerative illness defined by tau aggregates and neurodegenerative changes attributable to mutations in MAPT gene that encodes for the tau protein (Samudra et al., 2023). Some of the lines of evidence point toward existing supportive tau pathology in AD due to mutations in tau in specific forms of FADs. These mutations occurring in FAD highlight the genetic basis of tau-associated neurodegenerative pathology and central involvement in the pathogenesis of AD (Lansdall, 2014).

### 3.3 Alterations in synaptic functions and neurotransmission inhibition

The cholinergic system functions significantly in cognitive ailments and is involved in several forms of dementia (Figure 2). Cholinergic neurons with the nucleus basalis of Meynert secrete amyloid plaque and NFTs that lead to degeneration with pro-inflammatory events. Cholinergic deficiencies compromise the BBB and cause inappropriate transport of metabolites and failure to remove amyloid plaque (Ferreira-Vieira et al., 2016; Chen et al., 2022). Alterations in Ca^2+^ permeable n-acetylcholine-receptor (nAChR) may damage synaptic integrity (Shen and Yakel, 2009). Aβ binds with high affinity to α7-and α4β2-nAChRs which decreases the expression of choline acetyltransferase while increasing the activity of AChE. This reduction in ACh levels contribute to the progression of dementia (Roberts et al., 2021). The degeneration of noradrenergic neurons located in the locus coeruleus also plays a role in cognitive deficits and neurodegenerative processes. Serotonin is additionally involved in the development of AD, as most of the patients lose serotonergic neurons with decreased levels of neurotransmitter (Weinshenker, 2018).

Serotonergic inputs from the midbrain raphe nuclei influence cortical plasticity and memory formation, which results in memory impairment (Akyuz et al., 2024). Glutamate is a glutamatergic neurotransmitter acting both on NMDA and AMPA receptors to maintain synaptic plasticity. Disruption of glutamate/glutamine metabolism has the potential to cause neuron depolarization and excitotoxicity, which subsequently causes neuronal damage (Danysz and Parsons, 2012; Figure 2). Aβ induces hypersensitivity of NMDA receptors and interrupts its activity regulation, resulting in excitotoxicity. Interactions between GABA and serotonin in dorsal raphe nuclei influence cognitive processes; antagonists of serotonin receptor (5HT-6R) are promising for increasing serotonin levels, thus reducing the formation of amyloid plaques. It is because there is disrupted inhibitory regulation of GABAergic neurons over glutamatergic and cholinergic neurons, which is associated with the synaptic damage observed in AD (Figure 2). An imbalance of any neurotransmitter can accelerate worsening of symptoms in AD (Takahashi et al., 2010; Nakamura, 2013; Akyuz et al., 2024).

#### 3.3.1 Pre-synaptic dysfunction via Aβ

In pathological conditions, Aβ aggregates in synapses resulting from abnormal build-up of the BACE1 which is a crucial neuronal β- secretase involved in Aβ production. This implies that critical functions of presynaptic terminals, such as axonal transport, synaptic vesicle cycling, and neurotransmitter release, are impaired (Hampel et al., 2021). Aβ oligomers are found to co-localize with axonal voltage-gated calcium channels, causing increased calcium influx and disrupting the fast axonal transport of essential factors like BDNF (Gan and Silverman, 2015). Aβ can interfere with axonal transport through mechanisms that do not rely on tau or destabilization of microtubules, involving factors such as NMDA receptor activation, casein kinase 2 or calcineurin (Vossel et al., 2015).

Recent research has shown that when Aβ oligomers bind to the distal axons, it triggers local production of the transcription factor ATF4 within the axon, which then retrogradely translocate to the soma and contributes to neuronal degeneration (Walker et al., 2018; Salvadores et al., 2020). Presynaptic Aβ disrupts normal physiological axonal transport and induces pathological axonal transport, thereby facilitating retrograde spread of AD pathology (Dan and Zhang, 2023). Neurotransmitter-containing synaptic vesicles are essential for transmitting trans-synaptic signals. Aβ has the ability to modify the release, cycling, recycling and trafficking of these vesicles. This is characterized by oligomeric Aβ initiated presynaptic calcium influx enhances vesicle release, leading to high levels of glutamate and augmented spontaneous postsynaptic activity (Perdigão et al., 2020; Rivera et al., 2023). APP dimers located at the presynaptic plasma membrane functions as Aβ receptors, which enhance its production and induce synaptic hyperactivity. Aβ also impairs the recycling of vesicles through a decrease in the recycle process and enhancement of resting pool activity (Fogel et al., 2014). Recovery of the vesicle pool size may be possible through inhibition of CDK5 inhibitors. D-Serine, a co-agonist of NMDA receptor, is increased in brain regions, such as cortex, cerebrospinal fluid and hippocampus of AD patients. Increased D-serine within the cerebrospinal fluid is controversial since Aβ binding causes an increase in extracellular D-serine. A loss of APP correlates with lower extracellular D-serine levels, higher total D-serine levels, and diminished dendritic spine dynamics. Inhibiting pathological APP cleavage is crucial for maintaining D-serine balance and cognitive functions (Orzylowski et al., 2021; Ni et al., 2023).

#### 3.3.2 Post-synaptic dysfunctions via Aβ

Aβ oligomers are highly selective molecules that interact with the post-synaptic areas of excitatory synapses, altering their structure, composition and functionality. They may well be mediated by binding to cell surface molecules; increased synaptic excitotoxicity accompanies formation of Aβ receptor complexes. The mechanisms responsible for Aβ’s initial targeting and binding to synapses remain largely unknown, as does the degree of specificity of various Aβ species for different receptors or cell types (Dinamarca et al., 2012; Ding et al., 2019).

#### 3.3.3 Post-translational tau modification

Tau is one of the key microtubule-associated proteins that hold the microtubules together in neurons. The protein experiences various kinds of post-translational modifications, including phosphorylation, glycosylation, acetylation, nitration, glycation, ubiquitination and SUMOylation. The improper phosphorylation triggers the structural conformation of tau and later into NFTs (Guo et al., 2017). Phosphorylation is the most prevalent modification of tau, which can occur at serine/threonine or tyrosine or even both sites (Alquezar et al., 2021). One of the potential biomarkers of AD, phosphorylated tau at threonine 231, may be increased tau phosphorylation in cerebrospinal fluid and perhaps is the difference between AD and other types of dementias (Holtzman, 2011). Multiplicity of sites of tau protein which is phosphorylated by PDPKs or non-PDPKs. The major PDPKs that have been identified to be responsible for the tau protein phosphorylation are GSK3, CDK5, and ERK. Alterations in CDK5 activity are associated to the development of AD, resulting in a decline in dendritic spines and impaired synaptic plasticity, which leads to hyperphosphorylation of tau (Martin et al., 2013). GSK-3 is implicated in abnormal tau phosphorylation and should therefore be pre-phosphorylated on its substrate (Hooper et al., 2008). Research indicates that tau in the earlier stages of AD is acetylated which hampers cognitive activity and affects memory-associated proteins, activity- dependent cytoskeletal dynamics, and AMPA receptor trafficking, ultimately disrupting synaptic plasticity and memory performance (Tracy and Gan, 2017). In addition, enhanced NO signaling via glyceraldehyde -3- phosphate dehydrogenase (GAPDH) in the brains of AD patients shifts the balance toward tau acetylation, and inhibition of GAPDH-mediated NO signaling can help alleviate Aβ induced cognitive deficits (Sen et al., 2018).

#### 3.3.4 Pathological tau synaptic dysfunction

Pathological tau mutations alter the tau binding affinity and lead to increased abnormal aggregation, misplacement into different subcellular compartments and enhanced spread to other brain regions (Wu M. et al., 2021). AD pathologies lead to hyperphosphorylation of tau at pathological sites, release from microtubules, reduced stability of cytoskeleton and impairment in axonal transportation. Extracellularly localized oligomeric tau forms cause aberrant tau build-up in axonal regions and impede the axonal transport of membranous organelles (Rawat et al., 2022). Pathological tau associates with synaptic vesicles via synaptogyrin-3, thus destabilizing presynaptic functions (Largo-Barrientos et al., 2021).

A diffusion barrier linked to axon’s initial segment probably maintains the balance of tau distribution between axons and somatodendrites (Best et al., 2023). An inhibition of microtubule dynamics by Aβ could disrupt normal sorting of tau. A significant amount of tau in AD patients is relocated to postsynaptic compartments, altering the equilibrium between axonal and somatic-dendritic localization (Zempel and Mandelkow, 2014). Loss of microtubule integrity results in reduced synaptic clustering, excitatory synaptic transmission disruption, and memory impairments caused by the invasion of tau proteins into the neuronal dendrites (Yin et al., 2021). Transfection with truncated tau or its ablation reduced excitotoxicity caused by Aβ. Other approaches attempt to reduce the harmful impacts of synaptotoxicity in AD via the missorting targeting and specific phosphorylation at the postsynaptic compartment (Wu M. et al., 2021). AD Braak staging identifies the spread of NFTs with tauopathy, that could represent an endogenously propagated low-abundance species of high molecular weight tau. The trans-synaptic spread circuit-based from entorhinal cortex to hippocampus is facilitated by synaptic contacts and enhanced neuronal activity (Macedo et al., 2023). So, tauopathy in AD can be targeted by blocking tau propagation thus alleviating tau pathology.

### 3.4 Neuroinflammation

Neuroinflammation has been recognized as one of the critical mechanisms involved in the development of AD, significantly affecting the progression and severity of the condition (Kinney et al., 2018). Immune responses within the brain originate mainly due to an interaction between misfolded and aggregated proteins, like Aβ and tau, with their recognition through pattern recognition receptors on microglia and astroglia; this leads to the emission of inflammatory mediators and sustains a vicious cycle of neuroinflammation and neurodegeneration (Meraz-Ríos et al., 2013).

Microglia, resident immune cells in the brain, play a crucial role in the neuroinflammatory process associated with AD. In a normal scenario in the healthy brain, these microglia monitor their environment, or in a word, they maintain neuronal homeostasis (Nimmerjahn et al., 2005); however, in AD, through TLR2, TLR4, TLR6, and CD36, it is chronically activated by Aβ oligomers and fibrils, resulting in the release of pro- inflammatory cytokines such as IL1, IL6, and TNFα (Hickman et al., 2008; Michelucci et al., 2009; Leng and Edison, 2021). Other cytokines impair the integrity of dendritic spines and lead to microglial dysfunction in Aβ clearance. Genetic attributes, including TREM2 and CD33, also influence microglial function (Yu and Ye, 2015; Salter and Stevens, 2017). Activated astrocytes represent another type of cell implicated in neuroinflammation in AD (Avila-Muñoz and Arias, 2014). The interaction between them with neurons and microglia increases the inflammatory signals. Other cell types involved in the process are endothelial cells, oligodendrocytes, and neurons that produce immune molecules like IL6, ILβ, and CC motif ligand 2 (CCL2), once their integrity is compromised by Aβ plaque (Zhang W. et al., 2023).

The chronic activation of microglia does not permit them to clear out Aβ plaques while allowing them to continue producing pro-inflammatory cytokines, leading to an imbalance between pro-inflammatory and anti-inflammatory signals (Wang et al., 2023). NO synthesis is also increased in neurons and glial cells exposed to pro-inflammatory cytokines by the expression of inducible isoforms of NO synthase, resulting in increased Aβ aggregation and suppression of synaptic plasticity (Allaman et al., 2011; Meraz-Ríos et al., 2013). This is further compounded by systemic inflammation and metabolic conditions such as obesity due to the chronic nature of neuroinflammation present in AD (Ly et al., 2023). Systemic inflammatory signals can easily cross the BBB to enhance neuroinflammatory responses and promote acceleration in AD pathology (Xie et al., 2022). The loss of activated microglia’s inhibitory function due to the dysregulation of GABAergic system in AD is believed to contribute to the release of pro-inflammatory cytokines (Rivera et al., 2023). This proves that systemic health is significant in handling the disease and potentially slowing its progression in AD.

### 3.5 Oxidative stress

Oxidative stress is considered to be one of the key players in AD pathogenesis in imbalance between ROS and antioxidants. Consequently, oxidative damage is now reported to affect proteins, lipids and DNA, which leads to neuronal dysfunction and cell death. Aβ plaques and NFTs only increase oxidative stress further (Wang et al., 2014). Aβ can induce ROS generation through mechanisms such as NADPH oxidase activation, mitochondrial dysfunction and the vicious cycle of oxidative damage it perpetuates, and protein aggregation forms the pathology of AD (Leuner et al., 2012).

Mitochondrial dysfunction in AD is caused by impaired oxidative phosphorylation and increased levels of ROS. This leads to energy loss in the neurons, which creates even more oxidative stress (Aran and Singh, 2023). The primary contribution to oxidative stress in AD is made by genetics factors. Mutations in familial AD, particularly in the APP, PSEN1, and PSEN2 genes, result in an increase in Aβ production and accumulation (Dai et al., 2017) It has been noted that variations in genes responsible for antioxidant enzymes such as catalase, glutathione peroxidase, superoxide dismutase, influence an individual’s susceptibility to oxidative damage. Apart from the above-mentioned factors, other best identified genetic risk factor for AD includes APOE ε4 allele at high levels of oxidative stress and antioxidant defense levels (Vogrinc et al., 2023). Additionally, low levels of cytochrome C oxidase and hyperactivation of GSK-3 compromise mitochondrial permeability thus increasing ROS production (Zhang W. et al., 2023). Metal ions, particularly zinc and copper, chelate to Aβ plaques, therefore ROS might oxidize Aβ peptides and break cell membranes. Storage in the endoplasmic reticulum was blocked by the accumulation of Aβ plaques. This will be followed with increased cytosolic calcium and ROS (Bourassa and Miller, 2012). The overactivation of NMDA-type glutamate receptors (NMDARs) and NADPH oxidase leads to a production build-up that is ROS and reactive nitrogen species (RNS) (Ma et al., 2017).

Genetic variations in immune response genes, such as TREM2, influence microglial activity and the oxidative environment thus impairing microglial function and causing an increase in oxidative stress (Li et al., 2023). Antioxidant therapies have been examined in the context of AD treatment, and compounds like vitamin E, coenzyme Q10, and polyphenols have been shown promise in preclinical studies (Collins et al., 2022).

### 3.6 Genetic mutations

As discussed in the previous sections, genetic mutations are fundamental to the development and progression of AD. One of the most well-documented genetic factors involves mutations in APP, PSEN1, and PSEN2, which are closely linked to FAD. They result in aberrant processing of APP that leads to accumulations of Aβ plaques (Xiao et al., 2021). More importantly, the ApoE ε4 allele is a significant genetic risk factor for sporadic AD. The presence of APOE ε4 is linked to an increased load of Aβ and tau pathology, which further worsens neuronal dysfunction and disease progression (Liu et al., 2013). Some functions that have been characterized include disruption of lipid metabolism and synaptic plasticity (Estes et al., 2021). More recently, studies suggest mutations in the TREM2 gene encoding a receptor expressed by microglia. Mutations in TREM2 impair microglial function, leading to reduced clearance of amyloid plaques as well as a pro-inflammatory state that tends to exacerbate neuronal damage (Li et al., 2023). These mutations highlight the involvement of neuroinflammation in AD pathogenesis and concentrate on possible therapeutic interventions based on modulation of microglial activity. These insights into genetics advance our understanding of the mechanisms underpinning AD pathology and open up the possibility of targeted interventions.

## 4 Current therapeutic approaches for AD

**FIGURE 3:**
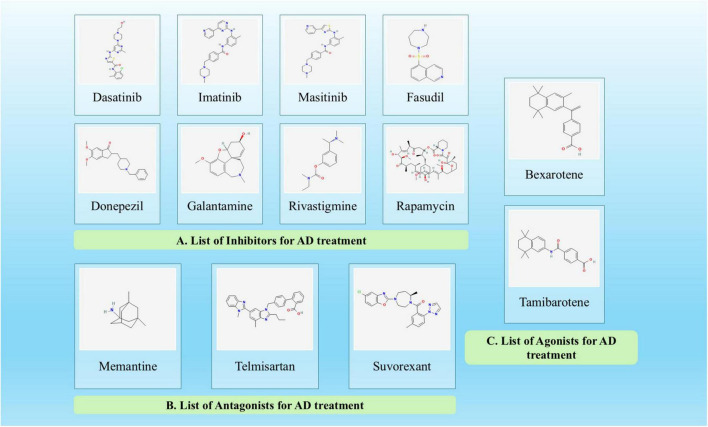
Chemical structures of key pharmacological agents in Alzheimer’s disease (AD) treatment. The figure illustrates the chemical structures of all the **(A)** inhibitors, **(B)** antagonists, **(C)** agonists that are involved in AD therapeutic strategies. The structures highlight the diverse chemical scaffolds being explored for AD therapeutics.

When developing medications to treat the condition, the most common focus has been on aging-induced neuronal death that lowers acetylcholine levels. The majority of current treatments are symptomatic rather than therapeutic (Hampel et al., 2018). Figure 3 provides the structure of all the inhibitors, antagonists and agonists that are used in the treatment approach so that it will be easier to observe that only slight modifications are present in the drugs that are used for AD treatments. These compounds represent distinct drug classes including cholinesterase inhibitors, NMDA antagonists, and monoclonal antibodies, each with unique chemical scaffolds designed to target specific AD pathogenic mechanisms (Murphy and LeVine, 2010; Yiannopoulou et al., 2019). Also, Tables 1, 2 will give a detailed overview along with the classification of the inhibitors, agonists and antagonists involved in the therapeutic regimen of AD. The FDA has approved aducanumab, the first monoclonal antibody, to target amyloid beta plaques in the brain. Patients with high amyloid levels and early-stage AD have typically been prescribed it (Haddad et al., 2022). Clinical trial outcomes suggest that aducanumab’s ability to reduce amyloid plaques has been linked to a slower decline in cognitive abilities, as assessed by clinical metrices utilizing Mini-Mental State Examination (MMSE) and Clinical Dementia Rating-Sum of Boxes (CDR-SB) scores (Vaz et al., 2022). However, there is intense dispute surrounding its clearance due to disagreements over how to interpret the study results and therapeutic usefulness. Another FDA-approved monoclonal antibody for early AD is lecanemab. Though it works through a different mechanism than aducanumab, it also targets amyloid-beta. Lecanemab has demonstrated efficacy in reducing decline in cognition because it promotes clearance of amyloid out of the brain (Cummings, 2023). There is a possibility for adverse effects, including ARIA-E that is brain swelling but subjects on lecanemab had a slower progression of symptoms compared to those on placebo (Abdelazim et al., 2024).

**TABLE 1:** Detailed overview of the inhibitors targeting Alzheimer’s disease: current therapeutic regime.

S.no.	Category	Name + DrugBank ID	Description	Mechanism of action	Side effects	Approved for other disease	Approved for AD	Clinical trial stage for AD	References
1.	Tyrosine kinase inhibitors	Dasatinib DB01254	• Orally available • Role as Antineoplastic and immunomodulating agent.	• Crosses BBB • Inhibits Aβ-induced microgliosis via specific tyrosine kinase activity inhibition. • Binds to active and inactive ABL kinase domain with higher affinity than imatinib.	Limited serious side effects reported in studies.	Chronic myeloid leukemia, (FDA approved, 2006)	No	Phase II/Recruiting Phase NCT05422885 NCT04685590	(Talpaz et al., 2006; Porkka et al., 2008; Shah et al., 2008; Dhawan and Combs, 2012; Rodríguez-Agustín et al., 2023)
		Imatinib DB00619	• 2-phenylamino-pyrimidine derivative protein • Treat chronic myelogenous leukemia, gastrointestinal stromal tumors (GIST), and other malignancies • Inhibits amyloid plaque formation, validated in lipopolysaccharide-induced AD model in mice.	• Binds to ATP pocket in active site, inhibiting BCR-ABL protein. • Inhibits RTKs for PDGF and SCF, c-Kit. • Inhibits proliferation and induces apoptosis in GIST cells with activating c-Kit mutation (*in vitro*).	Severe side effects and poor brain bioavailability.	Chronic myeloid leukemia, (FDA approved, 2001)	No	Preclinical stage	(Cancino et al., 2008; Weintraub et al., 2013; Gardner et al., 2016; Husaini et al., 2017; Saka et al., 2021; Kaehler and Cascorbi, 2023)
		Masitinib DB11526	• Phenyl-amino-thiazole type TK inhibitor • Multiple pharmacological targets • Neuroprotective • Improves cognition	• Reduces microglial activation and Aβ signaling. • Promotes synaptic protection. • Inhibition of mast cell, microglia activity • Modulation of Aβ and τ protein signaling • Prevent synaptic damage	Edema, gastrointestinal (GI) toxicity, rash, diarrhea, nausea, vomiting, and metabolic problems.	-	No	Phase II/III Trials NCT00976118 NCT05564169 NCT01872598	(Piette et al., 2011; Li et al., 2020; Ettcheto et al., 2021; Dubois et al., 2023; Neha and Parvez, 2023)
2.	Rho Kinase Inhibitor	Fasudil DB08162	• ROCK (Rho serine/threonine kinase) inhibitor • Vasoconstriction and vascular restoration • Targets neuroinflammation, promotes neuroprotection and axonal regeneration in AD models	• Inhibits ROCK-II • Reduces Aβ deposition, tau phosphorylation, and inflammation. • Stimulates neurotrophic factors	No significant side effects reported in studies.	Cerebral vasospasm (Approved in Japan and China, 1995)	No	Phase II/ Recruiting Phase NCT06362707	(Sayama et al., 2006; Hou et al., 2012; Song et al., 2013; Yu et al., 2017; Collu et al., 2024; Al-kuraishy et al., 2025)
3.	Acetylcholinesterase (AChE) Inhibitors	Donepezil DB00843	• Reversible, non-competitive AChE inhibitor • Enhances cholinergic neurotransmission. • Improves cognition and behavior in AD. • Neuroprotective	• Inhibits AChE • Protects against glutamate excitotoxicity. • Reduces Aβ toxicity. • Increases brain perfusion. • Stop the hydrolysis of ACh in the Brain	Gastrointestinal (GI) discomfort, Nausea, anorexia insomnia, muscle cramps, bradycardia.	-	1st: 1996 2nd: 2014 (Donepezil + Memantine) 3^rd^: 2022	Phase IV Completed NCT02787746	(Cacabelos, 2007; Szeto and Lewis, 2016; Adlimoghaddam et al., 2018; Sharma, 2019; Sheikh and Ammar, 2024; Yaghmaei et al., 2024)
		Galantamine DB00674	• Tertiary alkaloid • Enhances nicotinic receptor sensitivity. • Inhibits AChE • Improves cognition in mild-to-moderate AD	• Inhibits AChE • Modulates nicotinic ACh receptors to boost cholinergic function. • Enhances ACh levels	GI problems, nausea, vomiting, dizziness, anorexia.	–	2001	Phase IV Completed NCT01054976 NCT01478633	(Sodhi et al., 2008; Prvulovic et al., 2010; Tan et al., 2014; Koola, 2020)
		Rivastigmine DB00989	• Carbamate derivative • Parasympathomimetic or cholinergic agent • Neuroprotective • Treatment of mild to moderate AD dementia	• Inhibits both AChE and BuChE isoforms. • Prevents ACh hydrolysis • Enhances cholinergic transmission. • Slow disease progression	GI issues, headaches, nausea, vomiting, anxiety, agitation.	Parkinson’s Disease (FDA approved, 2000)	2000	Phase IV Completed NCT01585272 NCT02989402	(Bailey et al., 2011; Birks et al., 2015; Marucci et al., 2021; Vecchio et al., 2021; Delbreil et al., 2022; Siddique et al., 2022)
4.	Immunosuppressant and mTOR inhibitor	Rapamycin (Sirolimus) DB00877	• Antifungal agent • Immunosuppressive and antiproliferative properties • Helps in prevent organ transplant rejection	• Binds to FKBP12 to form a complex that inhibits mTOR • Indirectly modulates PI3K/AKT pathway, reduces inflammation • Anti angiogenic • Anti-proliferative • T-cell Suppression	Immunosuppression related risks, hematological effects, delayed wound healing, oral ulcers, pulmonary toxicity, liver function abnormalities, GI issues	Prophylaxis of organ rejection (Renal transplant) (FDA approved, 1999) Lymphangioleio- myomatosis (FDA approved, 2015)	No	Phase II/ Recruiting Phase NCT06022068 NCT04629495	(Kaeberlein and Galvan, 2019; Querfurth and Lee, 2021; Hou et al., 2023; Cai et al., 2024)

**TABLE 2:** Detailed overview of the antagonists/ agonists targeting Alzheimer’s disease: current therapeutic regime.

S.No.	Category	Name + DrugBank ID	Description	Mechanism of Action	Side effects	Approved for other disease	Approved for AD	Clinical trial stage for AD	References
1.	NMDA receptor Antagonist	Memantine DB01043	• Uncompetitive NMDA receptor antagonist • Targets glutamate excitotoxicity • Lower doses can result into enhance synaptic plasticity in brain, improve memory • Neuroprotective	• Reduces calcium influx to protect neurons • Leads to the improvement of Alzheimer’s disease symptoms • Good CNS effects	Dizziness, headaches. constipation, shortness of breath Overdose: ECG change, bradycardia, increased blood pressure, stupor, psychosis, vomiting	-	Moderate to severe AD (FDA approved 2003)	Approved	(Kishi et al., 2017; Matsunaga et al., 2018; Balázs et al., 2021; Tang et al., 2023; Singh and Kumar, 2024)
2.	Angiotensin II Receptor Antagonist	Telmisartan DB00966	• Treat hypertension, diabetic nephropathy, and congestive heart failure. • AT1R blocker with antioxidant and neuroprotective effects	• Crosses BBB • Effect on AChE • Reduces neuroinflammation, Aβ clearance, inhibits tau phosphorylation • Improves cognitive and memory function	Minimal side effects reported, generally well tolerated	Hypertension (FDA approved 1998)	No	Phase II NCT02085265	(Kume et al., 2012; Noda et al., 2012; Kurata et al., 2014; Abo-Youssef et al., 2020; Khalifa et al., 2020; Fu et al., 2023; Kuber et al., 2023)
3.	Orexin Receptor Antagonists	Suvorexant DB09034	• Dual antagonist of orexin receptors OX1R and OX2R • Targets insomnia and sleep disturbances commonly found in AD patients	• Blocks endogenous orexin neuropeptides at OX1R and OX2R	Somnolence, dry mouth, headache, agitation, diarrhea	Insomnia (FDA approved 2014)	No	Phase II/ recruiting stage, Phase III completed NCT04629547 NCT02750306	(Winrow et al., 2011; Brasure et al., 2016; Roecker et al., 2016; Han et al., 2023; Lucey and Bateman, 2023)
4.	Retinoid Receptor Agonists	Bexarotene DB00307	• RXR agonist with anti-cancer properties • Promotes ApoE4 expression for Aβ plaque clearance • Neuroprotective for both AD and PD (in disease model)	• Stimulates RXR to enhance ApoE4 expression • Blocks Aβ synthesis by binding Bex to BACE 1 • Reduces astrogliosis and reactive microglia • Improves synaptic plasticity	Dizziness, toe blister, dry cough, high triglycerides, high cholesterol	Cutaneous T-cell lymphoma (FDA approved 1999)	No	Phase II NCT01782742	(Bomben et al., 2014; Ghosal et al., 2016; Zhu et al., 2020; Vidal et al., 2021; Liu et al., 2023)
		Tamibarotene DB04942	• Retinoic acid analog targeting RA receptors • Regulates gene expression in CNS development • Improves memory and cognitive impairment	• BACE1 inhibition • Anti-inflammatory via NF-κB pathway • Reduces synaptic loss • Antioxidant • Improves Aβ_1–42_ clearance • Inhibits τ phosphorylation	Not widely reported	Acute myeloid leukemia (AML) (fast track designation by FDA 2024)	No	Phase II NCT01120002	(Shudo et al., 2004; Kawahara et al., 2009; Fukasawa et al., 2012; Jiang et al., 2016; Qiao et al., 2021; Behl et al., 2022)

The combination of memantine, an NMDA receptor antagonist, with donepezil, a cholinesterase inhibitor, is present in Namzaric. Approved for mild to moderate AD, this combination targets two critical neurotransmitter pathways simultaneously to improve cognitive function (Koola et al., 2018). While memantine modulates glutamate activity to limit excitotoxicity resulting from excessive glutamate signaling, donepezil increases ACh by blocking its catabolism. This combination is established, based on clinical research, to be more beneficial than either medication alone to enhance cognitive function and activities of daily life (Folch et al., 2018).

Approved for all stages of AD, donepezil increases the concentration of ACh in the synapse by reversibly inhibiting AChE. It has been demonstrated to be effective in all stages of AD and provides symptomatic relief that can persist for months to years. Side effects include gastrointestinal complaints such as nausea and diarrhea (Cacabelos, 2007). Available in a transdermal formulation as well as an oral agent, rivastigmine offers many treatment options. Unlike donepezil, it is known to inhibit not only AChE but also butyrylcholinesterase (BuChE), meaning that its effect on cholinergic signaling might be more wide-ranging (Kandiah et al., 2017). Rivastigmine is also used for managing dementia associated with Parkinson’s disease (PD) as well as mild to moderate AD (Müller, 2007). Galantamine is indicated for the treatment of the same but not for PD. The drug has been demonstrated to improve cognitive functions and overall quality of life. It is an AChE inhibitor that selectively inhibits the enzyme to enhance cholinergic transmission by modulating nicotinic receptors (Marucci et al., 2021). Besides AD medications, engaging in mentally stimulating activities, regular physical activity, a balanced diet, tackling sleep issues and developing healthy sleep routines can also aid in preserving cognitive abilities and slowing down cognitive deterioration (Key and Szabo-Reed, 2023). Behavioral approaches such as cognitive-behavioral therapy (CBT) or behavior modification techniques can assist in addressing difficult behaviors, while psychosocial strategies, counseling, and support groups can offer coping mechanisms, emotional support, and education for those with AD and their caregivers (Nakao et al., 2021).

Though these treatments do not reverse or modify the pathophysiology of AD; however, they can ameliorate symptoms by a multimodal approach that uses registered drugs to impact amyloid pathology and cholinergic dysfunction (Peng et al., 2023). Since the effect is individual, while some may benefit with minor advantages, for others increases will be highly notable in terms of their cognitive performance. Beyond that, these medications adverse effects could make long-term maintenance difficult. Another essential element of AD pathophysiology is amyloid buildup, which serves as the foundation for novel treatments that target Aβ (Zhang Y. et al., 2023). However, the debate over their potential therapeutic benefits also highlights the need for more investigation into biomarkers that could be able to identify patients who would benefit most from these treatments.

## 5 Imperative need for effective treatments

The increasing incidence of AD becomes a main global health concern requiring urgent attention. In addition to that, an increase in life expectancy comes hand in hand with suffering from this debilitating neurodegenerative condition (Li et al., 2022). The disease progresses slowly, causing cognitive and functional decline, burdening patients, families, and healthcare systems worldwide (Dhana et al., 2022). Inability to have proper disease modifying treatments for changing diseases makes breakthrough approaches to therapeutics even more vital today (Buccellato et al., 2023). Current therapies for AD focus on alleviating symptoms rather than providing a cure, aiming to slow down the advancement of cognitive and behavioral and psychological symptoms of dementia (BPSD). The Pharmaceutical Research and Manufacturers of America (PhRMA), AD has no known cure, making it a key research frontier in medicine, a problem for the pharmaceutical industry, and a burden on society (Passeri et al., 2022). As described in the previous section, only 7 medications have been successfully released till date. Furthermore, around 146 anti-AD medications failed in clinical practice globally between 1998 and 2017 (Correia et al., 2021). Genetic, environmental, and lifestyle variables all impact AD incidence, with women having a higher prevalence than men due to their longer life expectancy.

The increasing prevalence of AD creates enormous public health issues, underlining the importance of effective prevention, early detection, and therapy strategies (Zhang X. X. et al., 2021). The best chance to postpone the progression of disease development-advantageously, retaining its cognitive functions-is indeed provided in early diagnosis and intervention. Yet all these matters demand an urgent and rapid acceleration of research that encounters difficulties in early diagnosis and the limited availability of effective treatments (Pais et al., 2020). However, there is no gold standard medicine to successfully treat or stop AD pathogenesis, and the basic causes of these events are poorly known.

## 6 Drug repurposing: concept and strategies

**FIGURE 4:**
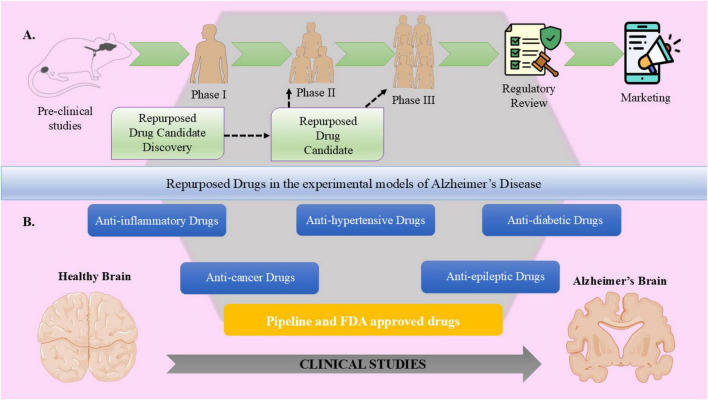
Drug repurposing for Alzheimer’s disease (AD). This diagram illustrates **(A)** drug repurposing process for AD, starting from preclinical investigations and advancing through all clinical trial phases, followed by regulatory approval and finally the marketing phase of the repurposed drug. **(B)** Different pharmacological drug classes, such as anti-inflammatory, anti-cancer, anti-hypertensive, anti-epileptic, and anti-diabetic compounds, which are investigated for their ability to have neuroprotective and disease modifying actions to modulate prominent clinical features of AD, such as neuroinflammation, oxidative stress, amyloid-beta (Aβ) accumulation, tau hyperphosphorylation, mitochondrial dysfunction, and synaptic loss, thus reverting brain health upon treating AD. The shift from experimental models to clinical applications is addressed, demonstrating how these various pharmacological classes can contribute to AD therapeutic strategies.

The process of developing drugs is intricate and expensive, often spanning several years of thorough research and costing millions of dollars. Before a candidate ligand can be put on the market, it must pass stringent clinical trials (Figure 4A; Cummings et al., 2019; Grabowska et al., 2023; Pan et al., 2024). Therefore, drug repurposing, also referred as drug repositioning, re-tasking or reprofiling, is a complementary *de novo* approach to traditional drug development, offering several advantages, including lower costs. Minimal risk of failure, less chance of unanticipated adverse effects or toxicity and reduced development timelines (Rao et al., 2023; Mag et al., 2024). For instance, thalidomide, which was globally banned in 1960s due to its teratogenic properties, was approved for treating leprosy in 1998 and for multiple myeloma as well in 2006. Drug repurposing involves the application of an already-approved medication or an unsuccessful candidate for a completely new indication that may differ from its original therapeutic indication (Chen et al., 2024). Basically, drug repurposing is one of the most effective and budget-friendly approaches in developing new drugs because it cuts the possibilities for further studies on safety and clinical trials. This approach is safer and faster in giving return on investment since it can be developed by the use of features from existing drugs (Ashburn and Thor, 2004; Pushpakom et al., 2019). By investigating the properties of current medications, researchers can uncover new therapeutic targets and pathways to address various medical issues. Additionally, experimental techniques based on targets and phenotypes, along with *in silico* resources like virtual high-throughput screening (HTS) and ligand- and structure-based molecular modeling, have become increasingly accessible for identifying candidates for repurposing (Wadood et al., 2013; Sadeghi and Keyvanpour, 2020; Panda et al., 2022; Gan et al., 2023; Rudrapal and Khan, 2023; Mohammadi et al., 2024). Drug repurposing streamlines the progression of laboratory research to the application of drugs. Patients who require new drugs sooner benefit from this process. Preclinical and very early-stage clinical research for repurposed drugs have already been conducted, thus streamlining the whole process (Krishnamurthy et al., 2022; Mag et al., 2024).

### 6.1 Historical successes in drug repurposing for other diseases

There are tremendous amounts of significant discoveries that made in the drug repositioning area. For example, Dimethyl fumarate, a drug for psoriasis repurposed by Biogen-Idec as an oral multiple sclerosis drug (Tecfidera) (Lategan et al., 2021). Various other examples from literatures highlight how drug repurposing has the potential to revolutionize therapeutic approaches, including those for AD as mentioned in the section 7. Researchers can accelerate the discovery of novel drugs and eventually improve the patients’ outcomes by utilizing clinical data and existing knowledge. Here are some examples of ancient yet well-known drugs that are repurposed for various other diseases.

Acetylsalicylic Acid, widely known as aspirin, is an example of drug repurposing, marketed as an analgesic for thousands of years. In the 1980s, it was repurposed as an antiplatelet aggregator to prevent cardiac events (Bohuon and Monneret, 2020; Fijałkowski et al., 2022). The analgesic and anti-inflammatory effects of aspirin are due to the inhibition of cyclooxygenase 2 (COX2), specifically vascular COX2, responsible for the production of prostaglandin. But this has harmful aftereffects on the gastrointestinal tract (Ornelas et al., 2017). Aspirin’s antiplatelet effects will be part of secondary prevention strategies to prevent brain strokes and secondary strokes (Xie et al., 2023). Its daily dosing can even prevent many cancers, most notably colorectal cancer, making it a drug that could soon be repurposed in oncology (Garcia-Albeniz and Chan, 2011; Rothwell et al., 2011). However, with intellectual property on this drug near expiration, the pharmaceutical industry has little incentive to help with repositioning.

Thalidomide, a notorious medication once prescribed for morning sickness, was prohibited by the World Health Organization (WHO) in the 1960s due to its ability to cause birth defects. However, it has shown significant efficacy in treating erythema nodosum leprosum, an autoimmune consequence of leprosy (Okafor, 2003; Kim and Scialli, 2011). In 1998, Celgene reintroduced it as an orphan drug for leprosy-related complications, but it is required to be used with stringent contraceptive precautions (Raje and Anderson, 1999). Because of the antiangiogenic activity of thalidomide, it was repositioned and became the first line treatment for multiple myeloma in 2006 (Vargesson, 2015) Thalidomide prescribing and dispensing in Europe are under specific monitoring (Jourdan et al., 2020).

Sildenafil was first explored by Pfizer in 1985 as a treatment for hypertension, demonstrating vasodilatory effects and preventing platelet aggregation by inhibiting phosphodiesterase type-5 (PDE5), the enzyme responsible for breaking down cGMP. However, during clinical trials in the UK, an unexpected side effect emerged, causing penile erections for which Viagra^®^ was marketed in 1998 in the indication of impotence (Ghofrani et al., 2006; Naylor and Schonfeld, 2014). Under this new indication, in 2005, Pfizer received approval for its third agent Revatio^®^ (Cavalla et al., 2017). Interestingly, it turns out that sildenafil works the same way for both erectile dysfunction and pulmonary arterial hypertension.

### 6.2 Innovative approaches and technologies in drug repurposing

The following section based on recent literature scores the invention in drug repurposing including high throughput screening (HTS), bioinformatics, artificial intelligence (AI), machine learning (ML) omics technologies, biomarker development and many more (Cha et al., 2018; Vora et al., 2023; Anokian et al., 2024). This method offers great benefits over traditional drug discovery (Papapetropoulos and Szabo, 2018). Nowadays advancement in technology and computational methods have remarkably accelerated the pace of drug repurposing research. Therefore, by utilizing existing knowledge and technologies scientists and researchers can identify novel uses for approved drugs and address unmet medical urgencies in AD and other likely diseases.

#### 6.2.1 Virtual screening

Virtual Screening (VS) has become a crucial method in drug development and repurposing efforts, as it performs efficient *in silico* evaluations across millions of compounds, ultimately enhancing the discovery of potential drug leads (Carpenter and Huang, 2018). The problem of VS data, when viewed as a computational challenge, can be framed as follows: input a ligand and receptor pair to compute their affinity values, aiming to minimize the number of compounds that require actual screening and to boost the efficiency of drug discovery and repurposing (Wu et al., 2024).

##### 6.2.1.1 Structure based virtual screening

Structure-Based Virtual Screening (SBVS), a type of method also known as receptor-based virtual screening, utilizes the 3D structure of target proteins (such as Aβ and tau) to forecast drug-protein interactions. This approach begins with the protein’s 3D structure and employs methods like molecular docking or molecular dynamics simulations to explore the distinct characteristics of the target protein’s binding site and its interaction patterns with small-molecule drugs. It assesses the binding potential between the proteins and drugs using an affinity scoring function (ASF) associated with binding energy (BE). Ultimately, drugs that receive high predicted scores are selected from a wide range of compound candidates for further bioactivity evaluation (Brooijmans and Kuntz, 2003; Fu et al., 2019).

Domperidone is an FDA-approved antiemetic drug is now considered a potential treatment for AD by targeting AChE, a key enzyme in AD therapy. SBVS was used to screen 1880 drugs approved by the FDA, and one of the promising AChE inhibitors was determined to be domperidone based on favorable binding interactions with the key residues of AChE. Domperidone was synthesized originally for gastrointestinal symptoms through its antagonism of the dopamine receptor. AD was repurposed as a prodrug that increased brain acetylcholine to enhance cognitive functions. Although BBB permeability is very low, using bioisosteric replacement techniques, analogs have been designed to improve BBB penetration without losing their inhibitory activity toward AChE. Therefore, this study demonstrates how SBVS facilitates identification, modifications already present within the drugs, and the new AD therapies with higher efficacy and better permeability for BBB (Goel et al., 2024).

##### 6.2.1.2 Ligand based virtual screening

Ligand-based virtual screening (LBVS) is a computational approach in which the structural characteristics of unidentified compounds are compared to those of known active molecules or ligands related to a target. Methods like similarity searching and pharmacophore modeling are utilized in this screening process (Singh et al., 2018; Ding et al., 2021). Nowadays, LBVS has proved to become one of the primary tools for drug repurposing against AD, targeting key proteins that are associated with AD such as Aβ-protein, GSK-3β, and Monoamine Oxidase B (MAO-B) (Speck-Planche et al., 2012).

LBVS utilizes highly characterized drug-like molecules toward discovering new inhibitors against proteins based on the similarity of molecules and predictive models. Flavones were also identified as inhibitors in the context of Aβ-protein, used through 3D-QSAR and docking experiments, showing high binding affinity as well as anti-aggregation activities needed for avoiding amyloid plaque formation (Yang et al., 2010). Similarly, GSK-3β inhibitors, including indirubin derivatives, have been optimized through their 3D-QSAR models to be specifically related with tau phosphorylation (Zhang et al., 2006). Many works have targetported with the use of LBVS methodologies for the search of new and more potent MAO-B inhibitors that targets neuroinflammation and Oxidative stress thereby improving cognitive symptoms in AD patients (Speck-Planche et al., 2012). LBVS, therefore, offers a resource-efficient path in repositioning existing compounds for AD, with applications expanding through complex machine learning algorithms and QSAR techniques that enhance predictive accuracy and broaden drug design against multi-target profiles in neurodegenerative therapy.

#### 6.2.2 Quantitative structure activity relationship modeling

The Quantitative Structure Activity Relationship (QSAR) model predicts the biological activity of molecular descriptors. The QSAR models, therefore, identify a potential drug candidate by that predicted biological activity. It establishes correlations between molecular properties and biological activities for the prediction of potency among the candidates. This computational method may identify repurposing candidates based on their AD activities that are predicted afterward (Tejera et al., 2020).

QSAR method emerged as an important tool for designing inhibitors for AD, particularly against the enzyme BACE1. Hence, BACE1 is a key component in amyloid plaque formation and thus has become a target drug for AD intervention (Das and Yan, 2017). Recently, QSAR models have been designed starting from different sets of molecular structures of datasets in order to predict the BACE1 inhibition capability. They include very diverse molecular descriptors that cover constitutional indices, 3D-MoRSE descriptors, and atom-centered fragments related to biologically useful properties (Ponzoni et al., 2019). By incorporating machine learning techniques and descriptor selection, QSAR models can reach accurate classification and predict the compounds’ inhibitory efficacy on BACE1. This approach simplifies filtration of possible drugs for AD out of existing molecules, thus providing a computationally efficient alternative for drug repurposing targeted at complex neurodegenerative conditions like AD.

#### 6.2.3 Machine learning and artificial intelligence

Machine Learning (ML) employs algorithms designed to explore large datasets to look for patterns that can predict drug activity. It can be further employed to rank candidates for repurposing based on considerations including target affinity, toxicity, and pharmacokinetics (Singh et al., 2023). Artificial intelligence (AI) is amongst the brightest technologies that can apply to both drug discovery and repurposing for AD. This allows for analysis of enormous data volumes that may contain new drug targets, novel design of new drugs, and predict efficacy and toxicity. The power also transfers AI repurposing algorithms that at present are applying existing drugs for AD treatment by analyzing their pharmacological properties and identifying new possible uses (Paul et al., 2021).

The integration of ML and AI has revolutionized the conventional drug discovery paradigms. These powerful technologies enable the analysis of huge biomedical datasets, encompassing molecular structure, gene expression profiles and pathology of various diseases (Yadav et al., 2024). By the identification of integrate pattern and relationships that may be failed to observe by conventional methods, ML and AI algorithms have significantly accelerated the discovery of new therapeutic applications for the existing drugs (Anokian et al., 2024). Such ML and AI algorithms as neural networks and deep learning models, for example, can be of good use for drug-target interaction prediction. Using such huge sets of training data as known interactions, such models generalize the predictions of novel drug-target pairs (Xu L. et al., 2021). This has proved crucial in identifying repurposable drugs for diseases such as AD, cancer, and other infectious diseases, and even COVID-19 (Cong and Endo, 2022; Vrahatis et al., 2023).

Furthermore, AI powered drug repurposing platforms like Drug Repurposing Hub (Corsello et al., 2017), DrugRepo (Wang et al., 2022), RepurposeDB (Shameer et al., 2018), and repoDB (Brown and Patel, 2017), along with various other web based databases that collectively enhance the drug repurposing field, have optimized the procedure by rapidly scanning and synthesizing data from diverse sources including scientific literature, clinical trial repositories and omics data (Cong and Endo, 2022). This opens potential therapeutic applications for existing drugs and hence the development of new treatment. These technologies enable identification of *de novo* target interactions, optimization of drug combinations, and prediction of drug efficacy along with toxicity, which may eventually translate into more effective and safer therapies for AD and other complex diseases (Vrahatis et al., 2023).

#### 6.2.4 High throughput screening and bioinformatics

High Throughput Screening (HTS) is an innovative approach for drug repurposing where screening of large libraries of compounds against AD-relevant targets (e.g., enzymes, receptors) to identify potential hits is done. It can be used to validate computational predictions and identify novel repurposing candidates (Szymański et al., 2011). Bioinformatics, a crucial component, analyses the massive data generated by HTS, identifying patterns and relationships between the components, primary molecular targets, diseases phenotypes and prioritize candidates of pre-clinical and clinical testing (Xia, 2017). This approach has proved valuable in identifying drugs with potential therapeutic applications in various neurodegenerative diseases like AD (Singh et al., 2022). Hence, by targeting specific pathways implicated in these disorders, researchers can repurpose drugs. The collaboration of HTS and bioinformatics offers a systematic and targeted approach to drug repurposing, reducing developmental time and cost and hence streamlines the drug discovery pipeline (Mishra et al., 2024).

#### 6.2.5 Omics technologies

Omics, including genomics, proteomics, and metabolomics, form the backbone of today’s repurposing efforts, also for AD (Chauhan et al., 2024). An illustration of a multi-step method to validate the aforementioned candidates is the Drug Repurposing for Effective Alzheimer’s Medicine (DREAM) study, which has highlighted the potential interest of STAT3 inhibitors and reopened the effectiveness of other compounds such as phosphodiesterase inhibitors. This methodology underscores the potential of repurposing strategies to facilitate therapeutic development in AD and address the current lack of effective treatments (Chauhan et al., 2024) A deeper understanding of disease mechanisms, the identification of biomarkers, and the detection of molecular targets responsive to pharmacological modulation are made possible by omics technologies, that offer a thorough understanding of biological systems at the molecular level. In precision medicine, where treatment is selected based on patient-specific molecular profiling, these data are very useful in omics (Hu et al., 2011).

Genomics has offered a view of the genetic associations of diseases, which enables researchers to search for appropriate drug targets (Sonehara and Okada, 2021). Drugs targeted for specific conditions whose pathways are known can now be targeted for diseases with the same or similar pathways (Nabirotchkin et al., 2020). Genetic associations have, for instance, proven helpful in repurposing statins originally developed for hypercholesterolemia for specific kinds of cancer and cardiovascular diseases (CVD) (Jiang et al., 2021). Proteomics is thus the determination of proteins expressed during a disease state, thus forming a background for the selection of drugs targeting these proteins (Cho, 2007; Mavridou et al., 2021) Proteomics has facilitated the identification of potential repurposing candidates for inflammatory diseases as well as AD by focusing on protein networks associated with disease pathology (Western et al., 2024). Metabolomics is the study of small molecules involved in cellular functions that provides information on how a drug affects metabolic pathways. Researchers have been able to repurpose drugs that can restore metabolic balance owing to its pivotal role in understanding the metabolic reprogramming associated with diabetes, cancer, and CVD (Pang and Hu, 2023).

According to omics approach, one study concludes that the intricacy of AD demands a change from a “one drug, one target” strategy to a network-based one. This entails focuses on interrelated endophenotypes such as tauopathy, neuroinflammation, and amyloidosis and employing multi-omics data and algorithms to provide personalized treatment options (Fang et al., 2020). As a result, combining multi-omics data enhances our understanding of disease processes, helps pinpoint drugs targeting disease-modifying pathways, and allows for the quick repurposing of existing medications, thus accelerating the discovery of treatments for various diseases, including AD.

#### 6.2.6 Biomarker development for drug efficacy and safety assessment

Biomarkers are the biological indicators of disease presence, progression or response to treatment. The researchers can assess drug efficacy, monitor disease progression as well as can predict the adverse drug reactions (ADR) (Califf, 2018). Personalized medicine and patient population satisfaction depend on the discovery of reliable biomarkers. Researchers can maximize treatment results and reduce ADR by determining which individuals are most likely to benefit from repurposed medication (Weth et al., 2023). For example, in neurodegenerative diseases like AD, biomarkers related to oxidative stress and inflammation have been used to choose drugs capable of modulating these pathways potentially alternating disease progression and hence working as a potential drug (Singh et al., 2019; Rekatsina et al., 2020). Additionally, to reduce ADR, researchers can pre-screen patient population and redesign the dosing strategies by identifying biomarkers that predict ADR (Wang et al., 2024). Therefore, biomarkers are an essential tool in drug repurposing as they provide valuable insights into disease mechanisms and drug responses, thereby aiding in the creation of more effective and safer treatments for AD and other intricate conditions.

Drug repurposing is an innovative approach to facilitate drug development process especially in addressing the unmet medical requirements and emerging health threats (Singh et al., 2020). The collaboration of the mentioned technologies has the potential to improve patient outcomes, make drug development more efficient and cost effective. As interdisciplinary collaboration grows, drug repurposing will remain a powerful tool in modern drug discovery.

## 7 Case studies on various repurposed drugs for AD

Several pharmacological drug classes initially designed to be employed for the management of cardiovascular, metabolic, psychiatric and other conditions are currently under investigation for their possible neuroprotective effects in AD (Figure 4B; Cummings et al., 2019; Grabowska et al., 2023; Pan et al., 2024). Case reports of repurposed drugs outline their multifaceted mechanisms of action, from inhibition of Aβ aggregation, decreased tau phosphorylation and modulation of neuroinflammation. This section presents an overview of key examples, which demonstrates the capability of drug repurposing to overcomes the failures of conventional drug discovery and provide new promise for effective treatments against AD.

### 7.1 Antihypertensive drugs

Hypertension, or elevated blood pressure, has been connected to neurodegenerative diseases like AD and cognitive deterioration. It is also a significant risk factor for various cardiovascular conditions. Aβ plaque buildup, tau protein tangles, neuroinflammation, and vascular dysfunction are all part of the pathogenesis of AD. Antihypertensive medications may have neuroprotective benefits beyond their basic function of controlling blood pressure, according to recent studies (Farnsworth von Cederwald et al., 2022). Antihypertensive medications such as losartan and nilvadipine assist prevent vascular damage that can result in cognitive impairment, and they also reduce vascular risk factors by treating hypertension. Furthermore, maintaining the health and function of neurons can be aided by increased cerebral blood flow, which improves cerebral perfusion (Tchekalarova et al., 2024). Antihypertensive medications also have anti-inflammatory effects; for example, losartan and nilvadipine both have anti-inflammatory qualities that can lessen the neuroinflammation linked to AD. Antihypertensive medications also help reduce oxidative stress; their antioxidant properties may shield neurons from oxidative damage, which is a major contributing factor to AD pathology (Nemati et al., 2011).

A dihydropyridine calcium channel blocker, nilvadipine has demonstrated promise in the treatment of hypertension and has been studied for possible advantages in AD. Vasodilation as well as decreased vascular resistance result from nilvadipine’s specific inhibition of L-type calcium channels. Besides reducing blood pressure, this step could enhance cerebral blood flow, which is important for preserving cognitive function (Lawlor et al., 2018). According to studies, patients with AD may benefit from nilvadipine. Nilvadipine treatment enhanced cognitive function and decreased amyloid-beta buildup in the brain, according to studies done on transgenic mice models of AD (Zhao et al., 2015).

The pathophysiology of AD is influenced by oxidative stress, which nilvadipine has been demonstrated to shield neuronal cells from Jinglong et al. (2013). When compared to a placebo, nilvadipine improved cognitive scores in a clinical trial examining its impact on people with mild-to-moderate AD (Saykin et al., 2015). The neuroprotective qualities of commonly used ARB losartan, which prevent calcium ions from entering smooth vascular muscle cells and cause vasodilation, have also been investigated. These drugs reduce the blood pressure by blocking the effects of strong vasoconstrictor angiotensin II causes vasodilation and lowering blood pressure by blocking angiotensin II activity at its receptor sites (Kjeldsen et al., 2002). Losartan also has anti-inflammatory qualities that could help with neurological diseases. Several studies have examined the potential of losartan for treating AD. Research has demonstrated that losartan administration can enhance cognitive function and reduce neuroinflammation in animal models of AD (Papadopoulos et al., 2017). Losartan may have neuroprotective effects through a mechanism that inhibits the generation of pro-inflammatory cytokines in microglial cells (Zhang et al., 2012). In observational studies, losartan has been linked to a decreased risk of dementia in hypertensive patients (Kehoe et al., 2021).

Repurposing hypertension medications like losartan and nilvadipine to treat AD is a potentially effective therapeutic approach. In addition to treating hypertension, their methods provide neuroprotective advantages that could enhance cognitive function in AD patients. More clinical trials will be necessary to determine these medications’ efficacy and safety profiles in the context of treating AD as research continues to clarify the intricacies of their effects on neuroprotection and vascular health.

### 7.2 Antidiabetic drugs

Insulin resistance and hyperglycemia are hallmarks of diabetes mellitus, especially type 2 diabetes (T2D), a chronic metabolic disease. In the last few years, there has been considerable focus on the connection between diabetes and neurodegenerative disorders, particularly AD. T2D patients are more likely to acquire AD, according to research, which raises the possibility that metabolic dysfunction and cognitive decline are related.

There is increasing interest in repurposing antidiabetic medications for the treatment of AD since insulin resistance and neurodegeneration share similar processes (Rohm et al., 2022). The drug that is most frequently used in this class is metformin. It mainly improves insulin sensitivity and reduces the quantity of glucose produced by liver. Metformin mainly lowers blood glucose levels via reducing hepatic glucose synthesis through a decrease in gluconeogenesis in the liver (Hunter et al., 2018). Metformin increases the absorption and use of glucose in peripheral tissues, especially in muscular tissue (Polianskyte-Prause et al., 2019). Furthermore, AMP-activated protein kinase (AMPK), crucial for regulating cellular energy balance, is stimulated by metformin (Zhou et al., 2001). Research has shown that metformin enhances cognitive function and reduces Aβ accumulation in animal studies (Lu et al., 2020).

One important aspect of AD pathogenesis, oxidative stress-induced apoptosis, has been shown to be prevented by metformin (Markowicz-Piasecka et al., 2017). According to a longitudinal research, metformin-using T2D patients were substantially less likely to acquire dementia than non-users (Chin-Hsiao, 2019). The medication group includes pioglitazone, which improves peripheral tissues’ sensitivity to insulin. The peroxisome proliferator activated receptor gamma (PPAR-γ), which regulates genes associated with glucose and lipid metabolism, is activated by pioglitazone (Di Marzio, 2008). Neurodegenerative disorders may benefit from their anti-inflammatory qualities (Zhang W. et al., 2023a). In transgenic mouse models of AD, pioglitazone has been demonstrated to improve cognitive function and reduce the formation of amyloid plaques (Fernandez-Martos et al., 2017). It has been demonstrated that pioglitazone reduces the buildup of amyloid plaque by suppressing the expression of BACE1 (Quan et al., 2019). Another study found that among those with T2D on metformin-based therapy, pioglitazone use was linked to a lower incidence of dementia (Ha et al., 2023).

### 7.3 Anti-inflammatory drugs

Targeting neuroinflammation is a promising AD treatment method. Anti-inflammatory medications suppress COX enzymes and may diminish Aβ formation. Anti-inflammatory drug may have antioxidant benefits, protecting neurons from the oxidative damage linked with the disease (Wong-Guerra et al., 2023). Ibuprofen is a non-selective NSAID that inhibits both COX1 and COX2 enzymes, resulting in lower levels of pro-inflammatory prostaglandins. This activity lowers inflammation and pain (Ju et al., 2022). Ibuprofen medication has been shown in studies employing transgenic mouse models of AD to lower Aβ levels and improve cognitive performance (McKee et al., 2008). Ibuprofen has been demonstrated to decrease the release of pro-inflammatory cytokines in microglial cells exposed to Aβ, implying its potential to reduce neuroinflammation (Blasko et al., 2001). A longitudinal study discovered that regular ibuprofen use was related to a lower risk of acquiring dementia among older persons (Blasko et al., 2001).

Celecoxib selectively inhibits COX2, an enzyme that is overexpressed during inflammation. Celecoxib reduces inflammatory mediator generation while sparing COX1 activity, which is essential for gastric mucosal integrity (Goldenberg, 1999). Celecoxib has been proven in animal experiments to decrease amyloid plaque accumulation and enhance cognitive function in mouse models of AD (Small et al., 2008). Celecoxib has been shown to suppress microglial activation and reduce pro-inflammatory cytokine release in response to Aβ exposure (Sánchez-Pernaute et al., 2004). Another study found that celecoxib administration was connected to better cognitive performance in individuals with mild cognitive impairment (Small et al., 2008). The potential of using anti-inflammatory medications like ibuprofen and celecoxib to treat AD is a viable route for therapeutic intervention. Their techniques not only treat inflammation but also provide neuroprotective advantages that may enhance cognitive outcomes in AD patients. As research reveals the intricacies of these drugs impacts on inflammation and neuroprotection, additional clinical trials will be required to demonstrate their efficacy and safety profiles in the context of AD treatment.

### 7.4 Antimicrobial/ antiviral drugs

Recent research indicates that neuroinflammation and infections might significantly contribute to the development and advancement of AD. Chronic inflammation in the brain can worsen neuronal damage, whereas infections can activate inflammatory responses that contribute to cognitive decline. This has prompted researchers to investigate the possibility of repurposing existing antimicrobial and antiviral medications for AD treatment. Antimicrobial medications cure infections caused by bacteria, viruses, fungi, and parasites (Van Eldik et al., 2016). Doxycycline and acyclovir are two important medications under investigation for repurposing in AD. Doxycycline, which is a broad spectrum tetracycline antibiotic, disrupts bacterial protein synthesis by attaching to the 30S ribosomal subunit. It also has anti-inflammatory capabilities via inhibiting matrix metalloproteinases (MMPs), which are responsible for tissue remodeling and inflammation (Samartzis et al., 2019).

Doxycycline treatment has been proven in transgenic mouse models of AD to lower Aβ levels and improve cognitive performance (Balducci et al., 2018). Doxycycline has been found to lower the production of pro- inflammatory cytokines in microglial cells exposed to Aβ, implying its potential to reduce neuroinflammation (Jantzie and Todd, 2010). A study discovered that long-term doxycycline treatment was linked to better cognitive function in patients with mild cognitive impairment (Ye et al., 2024). Acyclovir, an anti- viral drug, is used for treating viral infections, specifically herpes simplex virus (HSV). It inhibits viral DNA synthesis by selective phosphorylation by viral thymidine kinase, resulting in chain termination (Elion, 1982). Epidemiological studies have revealed that people with a history of HSV infections may be more likely to acquire AD. Acyclovir treatment has been associated with a reduced risk of AD in these patients (Lopatko Lindman et al., 2021). Acyclovir has been demonstrated to protect neuronal cell cultures against Aβ-induced cytotoxicity, indicating that it may play a role in lowering neurotoxicity associated with AD (Iqbal et al., 2020). Another study found that patients with a history of herpes simplex virus were less likely to develop dementia after using acyclovir (Young- Xu Y. et al., 2021).

### 7.5 Neuroprotective agents

Neuroprotective drugs are chemicals that assist protect neurons from damage or degeneration. Riluzole and lithium are two well-known neuroprotective medicines that have been studied for repurposing in AD (Sever et al., 2022). Riluzole is primarily used to treat amyotrophic lateral sclerosis (ALS). It acts by blocking the release of glutamate, an excitatory neurotransmitter that can cause excitotoxicity if present in excess. Riluzole may prevent neurons from injury by regulating glutamate levels (Blyufer et al., 2021). Riluzole has been proven in the transgenic mouse models of AD to lower Aβ levels and improve cognitive performance (Okamoto et al., 2018). Riluzole has been shown to protect neurons from glutamate-induced toxicity, a factor that is particularly significant in AD (Matthews et al., 2021). A study discovered that long-term usage of riluzole was related with improved cognitive abilities in individuals with mild cognitive impairment (Hascup et al., 2021).

Lithium is primarily employed as a mood stabilizer in bipolar disorder. Its neuroprotective benefits are ascribed to its capacity to inhibit GSK-3β (Li et al., 2002). Epidemiological studies have found that lithium medication may lessen the chance of acquiring dementia in persons with mood disorders (Ishii et al., 2021). In cell cultures subjected to hazardous stimuli, lithium has been demonstrated to inhibit Aβ formation while also promoting neuronal survival. Another study found that taking lithium lowered the risk of dementia in people with mood disorders (Shen et al., 2024).

## 8 Advantages and realistic prospects of drug repurposing in AD

As already mentioned in section 6, drug repurposing represents the strategic identification of new therapeutic uses for existing approved or investigational drugs (Recino et al., 2025). As AD prevalence rapidly escalates projected to affect over 50 million individuals worldwide by 2050 and with traditional drug development yielding limited therapeutic breakthroughs, repurposing offers a pragmatic alternative to conventional *de novo* drug discovery (Fatima et al., 2024). This approach has garnered substantial attention in AD research, with repurposed agents currently comprising approximately 33% of AD drug development pipeline, representing 46 compounds across 182 active clinical trials (Cummings et al., 2025).

### 8.1 Advantages of drug repurposing in AD

#### 8.1.1 Accelerated development timelines

One of the most compelling advantages of drug repurposing lies in its substantially reduced development timeline. Traditional *de novo* drug development requires 10–17 years from initial discovery to market approval, whereas repurposed drugs can potentially reach patients in 3–12 years (Krishnamurthy et al., 2022). For marketed agents that have already completed non-clinical studies and Phase I, II, and III trials for their original indication, development for AD can commence directly at Phase II or even Phase III, potentially saving 2.5–4 years of development time (Andrade et al., 2016). This temporal efficiency is particularly critical for AD patients and their families, for whom delayed treatment options can translate into irreversible cognitive decline (Liss et al., 2021).

#### 8.1.2 Reduced financial invested

The cost differential between traditional drug development and repurposing is striking. Developing a new molecular entity from conception to regulatory approval costs approximately $2.8 billion, while drug repurposing can achieve the same endpoint for an estimated $300 million (Pillaiyar et al., 2020). This 90% cost reduction stems from the elimination of redundant preclinical toxicology studies, Phase I dose-finding and safety trials, and extensive early-stage formulation development (Sun et al., 2022). The average cost of Phase I AD studies alone has been calculated at $79 million expenses entirely circumvented when advancing repurposed therapies (Cummings et al., 2022).

#### 8.1.3 De-risked safety profile

A major advantage of repurposed drugs is the comprehensive understanding of their safety, tolerability, and adverse event profiles established through prior clinical use (Malik et al., 2022). For AD, a disease affecting predominantly elderly populations with multiple comorbidities and polypharmacy, this pre-existing safety knowledge substantially mitigates the risk of unforeseen toxicities (Sutanto, 2025). The approval rate for repurposed drugs that have successfully completed Phase I trials reaches approximately 30%, a marked improvement over the less than 10% success rate characteristic of traditional NME development (Wu S. S. et al., 2021). This enhanced probability of success fundamentally alters the risk-reward calculus for pharmaceutical investment in AD therapeutics (Michoel and Zhang, 2023).

#### 8.1.4 Established pharmacokinetics and pharmacodynamics knowledge

Repurposed drugs benefit from well-characterized pharmacokinetic profiles, including absorption, distribution, metabolism, and excretion parameters (Low et al., 2020). This existing knowledge facilitates rational dose selection for AD trials and reduces uncertainties regarding drug-drug interactions a particularly salient consideration given that AD patients frequently receive cholinesterase inhibitors, memantine, and medications for comorbid conditions (Schneider, 2011). Furthermore, for many repurposed candidates, formulations optimized for patient compliance and manufacturing processes scaled for commercial production are already established, eliminating additional development hurdles (Bashir et al., 2023).

## 9 Challenges and limitations in drug repurposing

**FIGURE 5:**
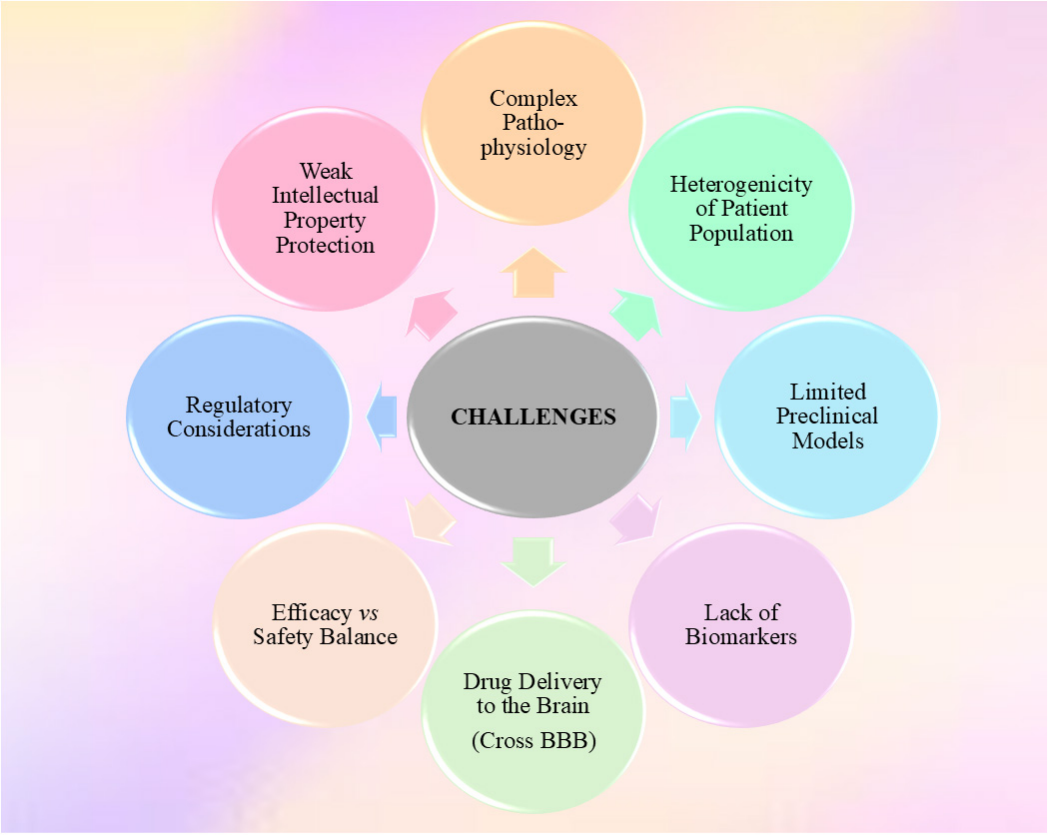
Major challenges to the development of effective AD therapies. These include complex pathophysiology of AD, characterized by variability among patient populations, the inadequacy of existing preclinical models to completely recapitulate the human condition, the absence of good biomarkers for early detection and disease monitoring tracking, difficulties in achieving adequate drug delivery through the blood-brain barrier (BBB), the challenge of balancing safety and efficacy in therapeutic manoevres, regulatory issues, and poor intellectual property protection for drug repurposing campaigns.

Drug repurpose strategy, to treat AD, could be considered a leap forward in the attempts to find effective interventions. While this strategy has some advantages, it is laden with numerous problems and constraints which if not dealt with, will limit its effectiveness and scope of use (Figure 5). In general, we can say, drug repurposing, while presenting a compelling strategy for AD therapeutics, encounters significant inherent limitations that constrain its efficacy as a monotherapy approach (Mishra et al., 2024). The fundamental challenge lies in the fact that most repurposed drugs were originally designed for single therapeutic targets in non-neurological conditions, rendering them often suboptimal for addressing AD’s multifaceted pathology (Singh, 2024). For instance, antihypertensive agents, though they demonstrate neuroprotective potential through reduced neuroinflammation and oxidative stress, address only discrete aspects of AD pathogenesis while leaving other critical mechanisms such as amyloid aggregation or tau hyperphosphorylation largely untouched. Consequently, relying solely on repurposed drugs may result in incomplete disease modification and limited cognitive benefits for patients (Jurcău et al., 2022). Additionally, the pharmacokinetic properties of repurposed agents may not be optimized for CNS delivery, particularly regarding blood-brain barrier penetration (Neumaier et al., 2021). While drugs like ibuprofen and doxycycline demonstrate promise in preclinical studies, their clinical effectiveness in AD is often constrained by inadequate brain bioavailability, requiring higher systemic doses that increase adverse effect risks (Cole and Frautschy, 2010). Furthermore, drug repurposing necessitates extensive clinical validation in AD populations, as the disease context markedly differs from the original therapeutic indication (Kulkarni et al., 2023). The heterogeneity of AD patient populations encompassing varying genetic backgrounds, comorbidities, disease stages, and rates of progression complicates the extrapolation of repurposing data from other diseases and undermines the development of universal treatment paradigm (Duara and Barker, 2022). The regulatory pathway for repurposed drugs also presents obstacles; regulatory agencies demand robust evidence of efficacy specifically in AD rather than mere extrapolation from historical use in other conditions (Saranraj and Kiran, 2025). These cumulative limitations underscore the necessity for either optimizing repurposed drugs through chemical modification and combination therapies or developing novel compounds specifically engineered to target multiple AD pathogenic pathways simultaneously, thereby enhancing therapeutic outcomes beyond what current repurposing strategies alone can achieve (Xia et al., 2024).

Moreover, available therapies tend to work on certain pathways or symptoms rather than the disease which most times is multi-faceted (Zhan et al., 2022). For example, drugs like donepezil, which are cholinesterase inhibitors, increase levels of acetylcholine but do not change the progression of the disease itself (Marucci et al., 2021). The complexity in the various biological systems makes it hard to find drugs to reposition for the treatment of AD that will target several fronts of the disease simultaneously. Apart from that, the problem is exacerbated by the limited knowledge of AD pathogenesis which hampers the predictions on which of the available drugs may modify the progression of theretofore existing disease (Gong et al., 2022). The degree of variation within the AD patient populations further poses challenges in the repurposing of drugs. Patients diagnosed with degenerative adenocarcinoma experience a multitude of symptoms and rates of disease progression that vary due to several factors such as age, other illnesses (such as heart-related conditions), and their lifestyle (Cha et al., 2018). This range contributes to the failure of drug developers in providing a universal treatment regime. As previously shown in numerous publications, if understood and ignored this variation may also produce varying outcomes in controlled clinical trials.

Hence, awareness of patient stratification and precise drug administration is imperative in any AD drug repurposing undertaking (Sun et al., 2022). It is critical to start with well-designed preclinical models of the disease in question, as is the case with potential drug repurposing medicine, prior to going into the clinical trials. Yet, most of the presently utilized models are incapable of reproducing the intricate pathophysiological changes seen in AD. The majority of the preclinical animal research models have narrow focuses rather on areas like amyloid plaques and modeling other aspects of neurodegeneration and dementia (Kulkarni et al., 2023). Besides, a lot of pre-clinical research involves either cell lines or animal models, which do not integrate well with human biology and a realistic representation of disease kinetics. This may lead to erroneous results in animal experiments that are not realistic for the human population (Loewa et al., 2023).

In this regard, it is critical to develop new and more sophisticated, effective and predictive preclinical models that can address the complex nature of the AD and evaluation of the efficacy of repurposed drugs. Lack of suitable tests for early diagnosis or for assessment of disease progression remains one of the great problems in drug repurposing for AD (Krishnamurthy et al., 2022). The possibilities offered by the adoption of biomarkers are huge as they may enhance understanding of the disease process and help in the selection of the right candidates for the clinical trial. Nevertheless, available modalities and standards like, amyloid-PET imaging or cerebrospinal fluid studies, are costly and quite invasive to many patients. It becomes hard to define the subset of patients that will be recruited for the clinical trials or evaluate properly the response to treatment in the absence of useful biomarkers (Bodaghi et al., 2023). Such a situation may pose a problem in identifying rapid acting medications that can be repurposed for specific patient populations. Hence the development of biomarkers which will be inexpensive and easy to perform will be paramount in challenges of drug repurposing in AD.

Achieving efficient delivery of treatment drugs across the BBB is still one of the major challenges encountered in addressing neurodegenerative diseases like AD. There are many possible therapeutics that could be repurposed, but the majority of them are incapable of crossing this barrier due to large molecular sizes or unfavorable chemical compositions. Consequently, although a drug may demonstrate effectiveness in preclinical studies or show benefits for different diseases, its efficacy in AD may be limited due to its inadequate capacity to cross BBB and reach necessary therapeutic levels (Iqbal et al., 2024). New mechanisms of delivery nanoparticles or nanocarriers, for example, are being developed to improve drug transport across the blood-brain barrier. Nevertheless, these technologies are currently in the initial phases of development and require substantial improvements before they can be used in routine clinical practice. The significance of the issue at hand, however, is linked to the need for addressing these barriers in the use of drugs which are set for repurposing (Cheng et al., 2023).

While certain medications may show promise in alleviating particular symptoms or in delaying the course of the illness under controlled conditions, their practical use may be hampered by management issues like taking multiple drugs or differences among patients. Such practice would require a thorough evaluation of safety especially when considering the “off-label” use of any drug by doctors of patients with AD (Lustberg et al., 2023). In most cases, regulatory bodies emphasize the need for strong evidence of the added advantages, if any, a repurposed drug will provide over existing ones, before giving their endorsement. This last requirement is one of the reasons repurposing drugs may be considered as a market risk for biopharmaceuticals as well as contribute to the lengthy timelines observed in the development process (Maziarz and Stencel, 2022).

### 9.1 Strategies for overcoming challenges

In order to counter such challenges and improve the likelihood of drug repurpose for AD, it is important to explore various approaches, including employing rigorous preclinical screening processes assists in the effective identification of potential candidates. This involves the application of sophisticated modeling systems such as organ-on-chip that closely represent human states and conditions (Low et al., 2020). Such systems might provide a better assessment of how efficacious and safe a specific substance is likely to be in the course of clinical development. Strategies based on personalized medicine will allow physicians to develop treatment plans for specific patients—differentiated by, for instance, genome or biomarkers—resulting in better efficacy of drugs with lower rates of adverse events. To optimize the clinical trial design and increase the overall probability of success, researchers target specific subpopulations who are most likely to respond to certain treatments (Ho et al., 2020).

The use of combined treatment with many drugs directed toward various pathological features of AD would likely be more effective than using only one drug. Combination therapies may also utilize current medications along with new candidates through repurposing which could improve total effectiveness while decreasing the risks associated with the use of single agents (Kabir et al., 2020). The therapeutic usefulness of treatments can also be studied in terms of real-world evidence using aforementioned data sources such as electronic health records and observational studies, rather than exclusively under controlled conditions as in clinical trials. Integration of real-world evidence (RWE) in research will seek to enhance the results of clinical studies while also providing considerations for more pragmatic-type studies in the future (Horton et al., 2021). This strategy would enhance the rates of participant recruitment if a trial explicitly indicated that the main purpose is to consider patients’ preferences regarding the treatment and outcomes while ensuring the achievement of the trial objectives. Involvement of patients in research not only elicits better compliance to the requirements but also results in the development of more meaningful studies aiming at improving quality of life alongside the primary endpoints (Mills et al., 2011).

**FIGURE 6:**
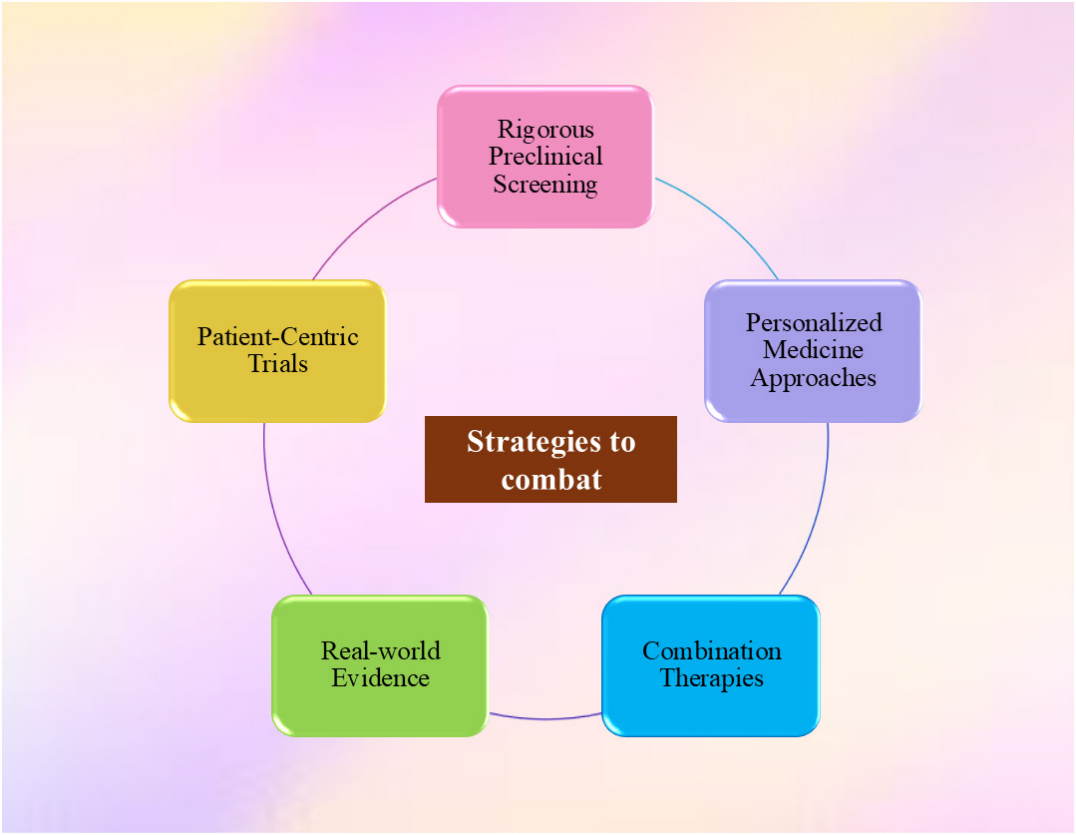
Innovative approaches to address the challenges identified and expedite AD drug discovery. These include rigorous preclinical screening for better potential translational value of drug candidates, approaches toward personalized medicine factoring in heterogeneity among patients, combinatorial regimens to target several pathogenic pathways, invest real-world evidence (RWE) to inform clinical trial design and treatment effect, patient-centric trials concentrating on outcomes that matter to patients and caregivers.

The challenges of the drug repositioning in AD, though complex, can be dealt with overcoming those issues such as: complexity of pathophysiology, heterogeneity of patients, limitations of any preclinical modeling, absence of suitable biomarkers, problems of delivery across the blood brain barrier, safety/efficacy implementation *vis-à-vis* regulatory restrictions and intellectual property issues, researchers can continue to search for new treatments for various diseases by using different techniques, such as personalized medicine in combination with extensive implementation of clinical trials using data from various populations in different periods of the trial. Figure 6 provides a general overview of the strategies that are needed to combat the challenges associated with drug repurposing.

## 10 Future directions and conclusion

AD is extremely complex because of the pathogenesis originating from multifactorial pathways; therefore, drug repurposing may be the promising strategy targeting multiple mechanisms of action via the application of known drugs for new indications. The future research should consider better assimilation of state-of-the-art technology like AI, ML, and omics approaches to drug discovery to quickly identify specific biomarkers for early diagnosis and therapy monitoring in AD. Interventions aiming at neuroinflammation-the main driver of pathology in AD are sizeable movers toward potential intervention. Anti-inflammatory drugs, immune modulators, and changes in lifestyle for systemic reduction of inflammation may slow the course of disease and improve outcomes. Drugs delivered across the BBB are a high-development area. Advancements in nanotechnology and molecular engineering will significantly enhance the bioavailability of repurposed drugs in CNS. Next-generation versions of such models, though obviously retaining the preclinical advantages, will provide even better recapitulations of human AD pathophysiology to allow for their more proper evaluation in terms of potential therapies. Biomarker-based patient stratification for personalized medicine approaches is necessary to ensure that interventions align each patient’s genetic and phenotypic profiles.

In conclusion, although current treatments offer only symptomatic relief, the most crucial step in unraveling this complex biology of AD is drug repurposing. Advanced computational tools, improved delivery mechanisms, and multifaceted therapeutic targets would be the stepping stones for innovative treatment. More importantly, the eradication of the barriers to AD therapeutics at present is critical, involving interdisciplinary research underlay by public-private partnerships in association with active community engagement. With ongoing investments and focus on research, the paradigm of AD treatment could shift from symptom management to disease modification. It holds hope to millions of individuals who suffer from this devastating neurodegenerative disease.
